# Click-code-seq reveals strand biases of DNA oxidation and depurination in human genome

**DOI:** 10.1038/s41589-025-02052-6

**Published:** 2025-10-31

**Authors:** Vakil Takhaveev, Nikolai J. L. Püllen, Navnit K. Singh, Lucie Lefevre, Emilie A. Aghajani, Sabrina M. Huber, Stefan Schauer, Hailey L. Gahlon, Anna R. Poetsch, Shana J. Sturla

**Affiliations:** 1 Department of Health Sciences and Technology, ETH Zurich, Zurich, Switzerland; 2 Functional Genomics Center Zurich, ETH Zurich, University of Zurich, Zurich, Switzerland; 3 Biomedical Genomics, Biotechnology Center, Center for Molecular and Cellular Bioengineering, Technische Universität, Dresden, Germany; 4 National Center for Tumor Diseases (NCT) partner site Dresden, German Cancer Research Center (DKFZ), Dresden, Germany

**Keywords:** DNA, Cancer therapy, Chemical modification, Bioinformatics, DNA damage and repair

## Abstract

DNA modifications drive aging, neurodegeneration, carcinogenesis and chemotherapy drug action. Accurate mapping of diverse DNA modifications with single-nucleotide precision in complex genomes remains challenging. We upgraded click-code-seq, a click-chemistry-aided DNA-modification mapping strategy, to enable its first application for sequencing oxidation and depurination in the human genome. We developed a companion fluorescence assay, click-fluoro-quant, to rapidly quantify common DNA modifications and novel adaptors to minimize false positives and assess modification frequency. We uncovered that endogenous DNA oxidation in a human cell line mirrors cancer mutational signatures linked to oxidative stress. The chemotherapy drug irofulven preferentially induces depurination in ApA dimers and promoters. Notably, oxidized guanines and apurinic sites, both irofulven induced and endogenous, are depleted in gene transcribed strands, with the strand bias increasing with gene expression. This work substantially advances click-code-seq for deciphering the impacts of key modifications in human DNA on cellular physiology and toxicological responses.

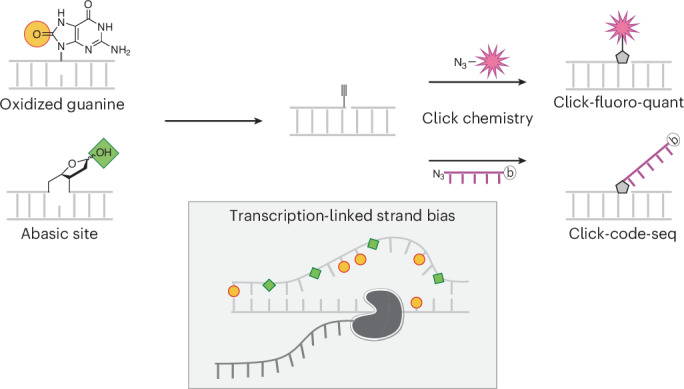

## Abstract (web summary)

By mapping oxidatively damaged bases and abasic sites at single-nucleotide resolution in human cells, Takhaveev et al. observed transcription-related strand biases, patterns mirroring cancer mutational signatures, and captured the action of the anticancer drug irofulven.

## Main

Genomic DNA (gDNA) can undergo chemically diverse modifications because of multiple endogenous and exogenous factors. Apart from enzymatically imprinted epigenetic modifications, the most abundant endogenous modifications include DNA breaks, abasic sites (AP sites), 8-oxoguanines (8-oxoG) and alkylation adducts, occurring at frequencies up to ~10^5^ per mammalian cell per day^1–3^. DNA modifications are key drivers of cancer (where they are mutation precursors^4^), aging^5^ and neurodegeneration^6^. Conversely, many anticancer drugs modify DNA to block replication and transcription in tumor cells^7^. Modifications such as breaks, AP sites and 8-oxoG are also emerging as epigenetic-like marks regulating gene expression^8–11^. Single-nucleotide localization of AP sites and guanine oxidation in large genomes remains largely unexplored because of technical challenges.

AP sites have been mapped at single-nucleotide resolution in human cells in two studies^12,13^, one of which also included mouse tissue analysis^13^. One reported guanine as the most frequently depurinated nucleotide^12^, while the other identified adenine^13^. The first study observed a stochastic genome-wide distribution of individual AP sites^12^, whereas the second reported recurrent single-nucleotide hotspots associated with sequence variants and, in mouse tissues, an accumulation in highly expressed genes, with a bias toward the nontemplate strand^13^. These discrepancies likely stem from methodological differences: snAP-seq involves AP site chemical tagging^12^, whereas SSiNGLe-AP involves endonuclease-mediated conversion of AP sites to 3′-OH termini followed by poly(A) tailing^13^. To the best of our knowledge, only one study has mapped 8-oxoG at single-nucleotide resolution with full coverage of a large genome, specifically in a human cancer cell line with and without potassium bromate exposure^14^. Therein, 8-oxoG was depleted in GC-rich regions and promoters, partly because of reduced 8-oxoG in G-quadruplexes^14^. The method used, CLAPS-seq^14^, combined chemical labeling of 8-oxoG from the 150-bp-resolution OG-seq assay^15^ with a DNA polymerase stalling strategy to mark modification locations^16,17^.

**Fig. 1:**
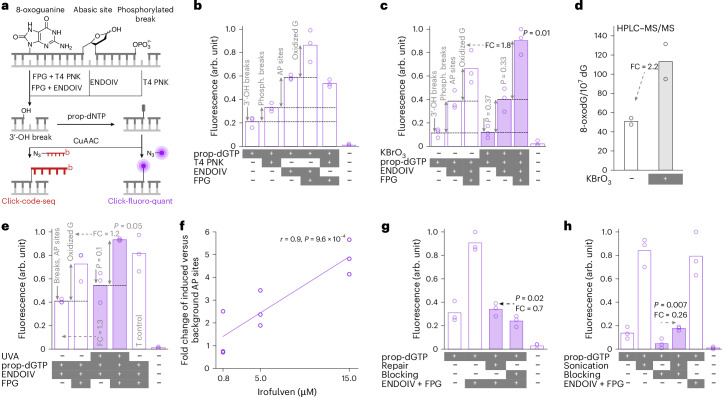
Click-fluoro-quant can be applied to quantify DNA oxidation, AP sites and breaks in gDNA. **a**, Click-code-seq: clicking DNA-modification sites with a code sequence followed by sequencing; click-fluoro-quant: clicking DNA-modification sites with a fluorophore followed by rapid fluorescence-based quantitation. DNA modifications are converted to 3′-OH-ended breaks by the indicated enzymes or preexisting 3′-OH-ended breaks are used. Alkyne-modified nucleotides (prop-dNTPs) are added to these 3′-OH sites. The alkyne is conjugated by copper click chemistry (CuAAC) with an azide-fluorophore or a sequencing adaptor called MoDIS (red oligonucleotide with b, a biotin group). **b**, Endogenous DNA modifications in human chronic myeloid leukemia (HAP1) cell gDNA measured using click-fluoro-quant. **c**, Click-fluoro-quant assessment of DNA modifications in HAP1 cells exposed to 50 mM KBrO_3_ for 30 min. **d**, HPLC–MS/MS quantification of 8-oxoG in gDNA of HAP1 cells exposed to 50 mM KBrO_3_ for 30 min. **e**, Click-fluoro-quant assessment of DNA modifications in BJ-5ta cells exposed to 10 J cm^−2^ UVA. T control, cells always staying in *T* = 37 °C incubator. **f**, Click-fluoro-quant assessment of induced AP sites in U2OS cells after 4-h exposure to irofulven. Markers, biological replicates (*n* = 3), each of which included three drug concentrations and a vehicle control to which the plotted values are normalized. The Pearson’s correlation coefficient (*r*) with respective two-sided *P* value is shown. **g**, Click-fluoro-quant assessment of repair and blocking strategies to remove background DNA modifications in HAP1 gDNA. Repair, nucleotide addition and ligation. Blocking, ddNTP insertion. Before both repair and blocking, 3′-OH groups were released from AP sites and phosphorylated breaks using ENDOIV. **h**, Click-fluoro-quant assessment of sonication-induced artifactual DNA modifications with and without ddNTP blocking in HAP1 gDNA. In **b**–**e** and **g**,**h**, the bar represents the mean of *n* = 3 (click-fluoro-quant) or *n* = 2 (HPLC–MS/MS) biological replicates (markers).The *P* values of a one-tailed (in **c**,**e**, H_A_ is greater; in **g**,**h**, H_A_ is less) paired *t*-test between the exposed and unexposed samples treated with the same set of enzymes (**c**,**e**) or between the arrow-connected samples (**g**,**h**). FC, fold change relative to arrow-indicated condition.

We previously developed the other single-nucleotide-precision method for guanine oxidation mapping, click-code-seq, and used it to characterize oxidation profiles in the yeast genome^18^, which is ~256 times shorter than the human genome. In click-code-seq, oxidized guanines are enzymatically converted to one-nucleotide gaps, which then are filled with prop-dNTPs (Fig. 1a). Resulting 3′-alkynyl DNA is tagged with a 5′-azido-modified code sequence by a copper(I)-catalyzed click reaction, generating a biocompatible triazole-linked DNA^19^ and allowing single-nucleotide-level detection of the original oxidation sites by DNA sequencing^18^. Consistent with data from human cancer cells (CLAPS-seq^14^ and 250-bp-resolution AP-seq^20^), oxidized guanines were depleted in yeast promotors^18^. This contradicted promoter enrichment observed for mouse cells^15,21^ and tissues^22^ and human noncancerous cells^21^, albeit at much lower resolution (over 100 bp)^15,21,22^. Overall, the few single-nucleotide-precision datasets on AP sites and guanine oxidation are inconsistent with each other and with lower-resolution measurements.

High-precision mapping of AP sites and guanine oxidation faces several challenges, such as distinguishing modifications from one another. To address this, in snAP-seq, aldehyde moieties of AP sites were selectively chemically tagged^12^ and, in SSiNGLe-AP, preexisting 3′-OH breaks were blocked using biotinylated ddCTP, enabling endonuclease conversion of AP sites into taggable 3′-OH breaks^13^. CLAPS-seq achieves high specificity for 8-oxoG by oxidation with K_2_IrBr_6_ and biotin tagging^14,15^ but its reliance on polymerase stalling may misidentify 8-oxoG sites because of stalls at AP sites in enriched DNA fragments. Secondly, DNA breaks, AP sites and oxidized bases can arise artifactually during sample preparation^23,24^. CLAPS-seq addresses this by labeling 8-oxoG immediately after DNA extraction^14^ but other methods include sonication or high temperatures before labeling^12,13^, potentially promoting artefactual depurination. Thirdly, spurious priming during DNA library amplifications can reduce accuracy, especially for low-abundance modifications in large genomes. Lastly, current approaches cannot distinguish whether a DNA modification occurs in one or multiple cells at a specific genomic site. These limitations likely contribute to the inconsistencies observed across different mapping studies.

Here, we comprehensively upgraded click-code-seq, previously used to map oxidation in yeast genome^18^ and bacterial plasmid^25^ DNA, to accurately locate oxidized guanines and AP sites in the human genome at single-nucleotide resolution. First, we developed a fluorescence-based assay click-fluoro-quant (Fig. 1a) to rapidly quantify DNA breaks, AP sites or guanine oxidation occurring endogenously or induced by representative stressors in human cell lines. Using click-fluoro-quant, we optimized a procedure to block background modification sites for click-code-seq analysis. Second, we designed a new type of sequencing adaptor to ligate target sites, distinguishing them from library amplification artifacts and assessing site-specific DNA-modification frequencies in cell populations. Having thus increased the specificity of click-code-seq, we mapped endogenous guanine oxidation and AP sites, both endogenous and induced by the chemotherapeutic drug irofulven, in human cancer cell lines. This work provides new insights concerning the genomic function of DNA modifications, with relevance to mutagenesis, DNA methylation, chromatin structure, DNA replication, transcription, DNA repair and the integrity of mitochondrial DNA (mtDNA).

## Results

### Rapid quantification of DNA modifications

**Extended Data Fig. 1:**
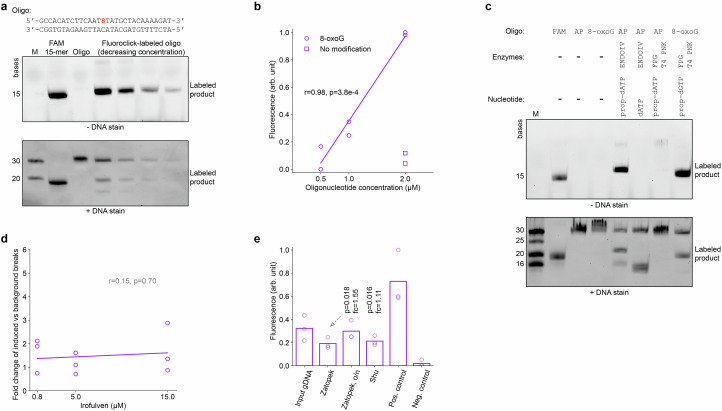
Click-fluoro-quant to assess DNA-modification levels in oligonucleotides and gDNA. **a** Products of click-fluoro-quant of 8-oxoG site at position 15 in the double-stranded 30-mer DNA oligonucleotide in a PAGE gel (experiment performed once). 8: 8-oxoG. Labeled product: AF594-labeled 15-mer fragment. The unmodified 30-mer complimentary strand and the unlabeled 15-mer fragment originating from 8-oxoG excision are visible in DNA staining. AF594 conjugation causes a higher retention time, resulting in a band corresponding to a strand of 20 bases. **b** Fluorescence intensity of click-fluoro-quant-labeled oligonucleotides containing 8-oxoG (**a**) and lacking this DNA modification. n = 2 independent (biological) experiments, each of which tested n = 3 concentrations of the 8-oxoG oligo. **c** Click-fluoro-quant of a 30-mer oligonucleotide containing an AP-site-mimicking tetrahydrofuran at position 17 or 8-oxoG at position 15 (**a**) in a PAGE gel (experiment performed once). **d** Click-fluoro-quant of irofulven-induced 3′-OH breaks in U2OS cells after 4-h exposure to the drug. Extracted gDNA was incubated overnight at 37 °C (to induce depurination of irofulven adducts, Fig. 1f) and labeled without enzyme treatment. Markers: biological replicates (n = 3), each of which included three drug concentrations and a vehicle control by which the plotted values are normalized. **e** Click-fluoro-quant comparison of gDNA repair efficiencies of three protocols: according to Zatopek et al.^29^ (used in Fig. 1g), Zatopek et al. with overnight (o/n) enzymatic repair and Shu et al.^30^ (Supplementary Note 1). In panels **a**,**c**, the gel was imaged in the fluorophore and DNA-stain channels before and after applying GelRed DNA stain, respectively; M, marker; FAM, FAM-labeled 15-mer oligonucleotide. **b,d**: r and p: Pearson’s correlation coefficient with respective two-sided p-value. In panel **e**, bar: mean of n = 3 biological replicates (markers); p: p-value of the one-tailed (H_A_: greater) paired t-test between the samples under the value and arrow-indicated; fc: fold change of DNA modifications relative to arrow-indicated condition; Pos. control: FPG and ENDOIV; Neg. control: dGTP instead of prop-dGTP.

To optimize library preparation for DNA-modification sequencing, we developed a simple fluorescence-based assay, click-fluoro-quant, for comparing quantities of DNA oxidation, AP sites and breaks. Using a double-stranded 30-mer DNA oligonucleotide with 8-oxoG at position 15, we converted the 8-oxoG to a dephosphorylated 3′-OH site with formamidopyrimidine DNA glycosylase (FPG) and T4 polynucleotide kinase (T4 PNK). We filled this site with 3′-(*O*-propargyl)-dGTP (prop-dGTP) using Therminator IX^26^ DNA polymerase and labeled it with AF594 picolyl azide by copper(I)-catalyzed azide-alkyne cycloaddition (CuAAC) (Fig. 1a). Fluorescence intensity from the resulting AF594-labeled 15-mer (Extended Data Fig. 1a) correlated linearly with 8-oxoG input concentration (Extended Data Fig. 1b). We followed the same approach for an AP site model by using a tetrahydrofuran-containing oligonucleotide, prop-dATP and endonuclease IV (ENDOIV) (Extended Data Fig. 1c).

We could distinguish and measure oxidized bases, AP sites and breaks in human gDNA, converting each modification selectively into 3′-OH groups for subsequent fluorescent labeling (Fig. 1a,b and Supplementary Note 1). We could quantify DNA modifications in cells exposed to representative stressors, namely, potassium bromate (Fig. 1c,d), ultraviolet A (UVA) irradiation (Fig. 1e) and the depurination-inducing chemotherapeutic drug irofulven^27,28^ (Fig. 1f, Extended Data Fig. 1d and Supplementary Note 1). Next, we used the approach to optimize conditions for minimizing the tagging of background DNA modifications that can confound sequencing of relevant modifications (Supplementary Note 1). We found that fewer unspecific 3′-OH groups were present after ddNTP blocking^18^ versus dNTP repair^29^ (Fig. 1g), even after optimization of the latter^30^ (Extended Data Fig. 1e). Lastly, to address artifactual modifications, we evaluated their levels associated with gDNA sample-handling factors, which suggested avoiding heat inactivation of enzymes (Supplementary Fig. 1a,b) and fragmenting gDNA by sonication, a well-established source for artifactual modifications^23^, only after both ddNTP blocking of background 3′-OH groups and labeling of the target DNA modifications (Fig. 1h, blocking versus sonication + blocking).

Click-fluoro-quant is, thus, a rapid (~3.5 h) and accessible assay for direct quantification of DNA strand breaks, AP sites and oxidative lesions, offering practical advantages over mass spectrometry (MS) despite potential fluorophore self-quenching effects. This method can be used to increase specificity in DNA-modification sequencing, as demonstrated also in recent mapping of *O*^6^-methylguanine induced by the glioblastoma drug temozolomide^17^.

### Precise sequencing of DNA oxidation in the human genome

**Fig. 2:**
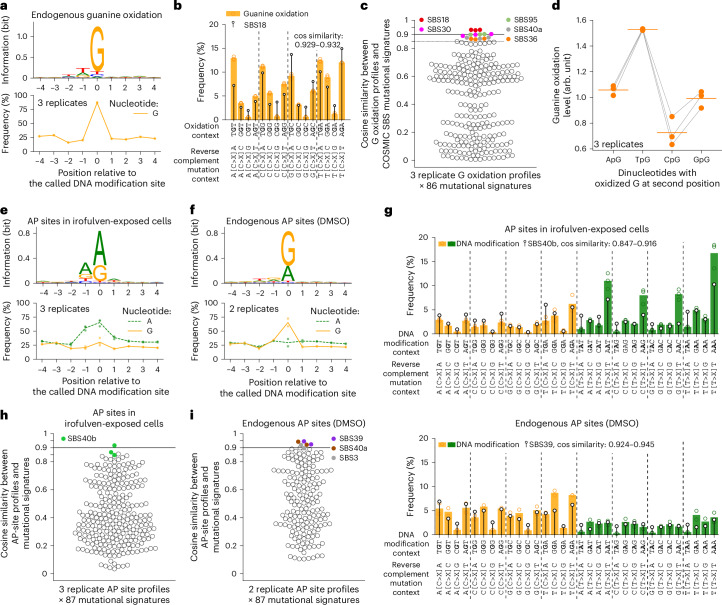
Click-code-seq uncovers single-nucleotide-resolution precursors of the ROS-related mutational signature SBS18 and identifies that irofulven induces depurination in adenine dimers. **a**, Sequence logo and guanine frequency in the local genomic context around called DNA oxidation sites in HAP1 cells. **b**, The distribution of endogenous guanine oxidation in trinucleotide contexts in HAP1 cells (*n* = 3 replicates) matched with the ROS-related mutational signature SBS18 from human cancers (COSMIC). **c**, The endogenous guanine oxidation profile has the highest cosine similarity to SBS18 among all 86 COSMIC SBS signatures. **d**, The endogenous guanine oxidation level is lower in CpG dinucleotide compared to other dinucleotides. Line-connected markers: biological replicates. Horizontal lines: means. The normalization of DNA-modification level is described in the Methods. **e**,**f** Sequence logo and frequencies of guanine and adenine in the local genomic context around called AP sites in U2OS cells exposed to irofulven (**e**) or DMSO (**f**). **g**, The distribution of AP sites regarding trinucleotide context in U2OS cells exposed to irofulven (top; *n* = 3) or DMSO (bottom; *n* = 2). The AP site profiles are matched with the indicated mutational signature. **h**,**i**, Comparison between the AP site profiles from U2OS cells exposed to irofulven (**h**) or DMSO (**i**) and 86 mutational signatures from COSMIC plus one experimental illudin S mutational signature^43^. In **a**,**e**,**f**, sequence logo: merged data of all biological replicates; markers and line: frequency in biological replicates and the mean, respectively. In **b**,**g**, markers and filled bars: frequency of a trinucleotide with a mapped modification of G (**b**) or G and A (**g**) in the second position shown for each replicate and as the mean, respectively; lollipop: mutational signature shown for C substitution (87% of SBS18) in **b** or for C and T substitutions in **g**; frequencies of DNA modifications and mutations each add up to 100%; X: A, G and T considered together. In **c**,**h**,**i**, marker: cosine similarity between one mutational signature and one replicate DNA-modification profile, which is calculated by matching a vector of 32 trinucleotide-specific 5′N[C/T>X]N3′ substitution frequencies and a respective vector of DNA-modification frequencies (for guanine oxidation, values corresponding to T>X substitution are set to zero); N: one of four nucleotides; X: A, G and T considered together.

**Extended Data Fig. 2:**
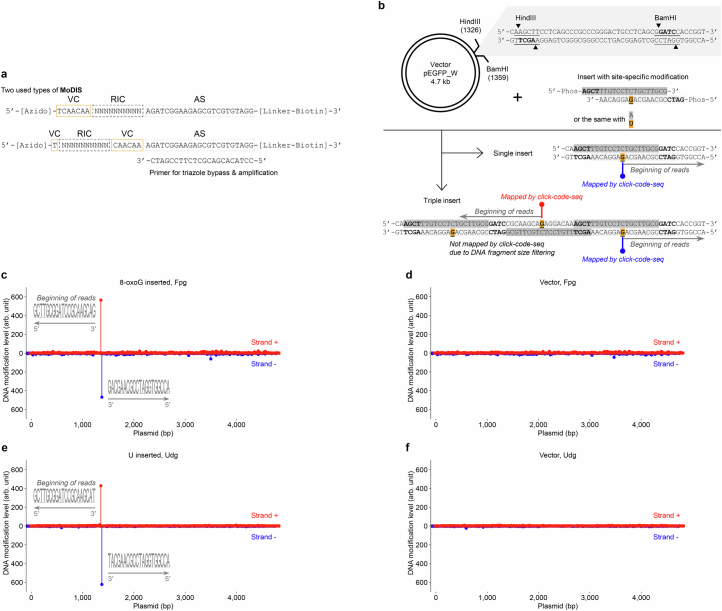
The upgraded design of the code adapter, MoDIS, and mapping site-specific DNA modifications in plasmid DNA. **a** Two used types of MoDIS. VC, validation code; RIC, randomized index code; AS, annealing site sequence. Details on improving click-code-seq specificity and sensitivity with MoDIS in Supplementary Fig. 3. **b** Plasmid construction with site-specific 8-oxoG (G) or uracil (U) via restriction-ligation strategy. In addition to a single insert, a triple insert can emerge by the ligation of three identical double-stranded oligonucleotides due to the complementarity of sticky ends and the excess of the oligonucleotides. If two DNA modifications are located closely in the same strand (for example, bottom strand of the triple insert), only the 5′-most one is mapped due to the size filtering ( ~ 200 nt) of the DNA fragment upstream of 3′-most DNA modification. **c-d** Levels of guanine oxidation along the plasmid DNA with inserted 8-oxoG (**c**) and the vector DNA (**d**). In **c**, plasmid DNA with inserted 8-oxoG ( ~ 25 fmol) was combined with a ~ 28-fold excess of vector. DNA was treated with Fpg, formamidopyrimidine DNA glycosylase, to recognize oxidized guanines, and prop-dGTP was incorporated in the gaps. **e-f** Levels of uracil (U) along the plasmid DNA with inserted uracil (**e**) and the vector DNA (**f**). DNA was treated with Udg, uracil-DNA glycosylase, to recognize uracil and converting it to an AP site, and prop-dTTP was incorporated in the gaps. **c-f**: in each panel, the experiment was performed once. DNA modification level (arb. unit) is relative to the median signal in the plasmid DNA. In panels **c**, **e**, we provide sequence LOGOs for the beginning of reads that identify the peak signals; the conserved bases match the expectation from the plasmid design (**b**). After cropping RIC and VC from the reads, the first 20 bases were reverse complemented, and the LOGOs were built using base frequencies (LOGO total height is 100% at each position).

An additional upgrade of click-code-seq involved creating a conceptually new version of the code sequence called a modified DNA identifier sequence (MoDIS). MoDIS is comprised of three modules, namely, a validation code (VC) of fixed nucleotide sequence, a randomized index code (RIC) and an annealing-site sequence (AS) for specifically binding a primer in PCR amplification (Extended Data Fig. 2a). We combined MoDIS and the ddNTP blocking of the background to generate single-nucleotide-resolution maps of endogenous DNA oxidation in gDNA from the human chronic myeloid leukemia cell line HAP1 (Supplementary Fig. 2). Using VC, we filtered out around 15% unspecific reads, likely amplification artifacts (Supplementary Fig. 3a-c). MoDIS increased the number of identified oxidation sites by around 35% as, without RIC, identical reads originating from different cells or genomes would have been removed by deduplication (Supplementary Fig. 3d,e). Most identified gDNA-modification sites (~90%) were guanines (Fig. 2a), which may be attributed to 8-oxoG and fapy-guanine^31,32^. These were supported by over 20 million unique reads (Supplementary Fig. 4, top, stars). The small portion of nonguanine sites (Fig. 2a) may be because of (1) FPG recognition of other modified nucleotides, for example, 8-oxoadenine, fapy-adenine, 5-hydroxy-cytosine, 5-hydroxy-uracil^31,32^ or 8-oxoG paired with A, T or G^31^ and (2) Therminator IX promiscuity and lack of exonuclease proofreading^26^, leading to prop-dGTP misinsertions. Lastly, as a control, we confirmed the successful calls of site-specific 8-oxoG in plasmid DNA (Extended Data Fig. 2b–d).

Concerning genomic site specificity, we identified that 13,574,049–14,065,470 (~90%) oxidized guanine sites were unique for each of three replicate analyses, whereas 259,973 (1.7%) single-nucleotide sites were oxidized in all three replicates (Supplementary Fig. 5a) and likely multiple cells within each replicate (Supplementary Fig. 5b). Yet, when the data were aggregated in 100-kbp bins, pairwise correlations were above 0.88 among triplicate analyses (Supplementary Fig. 3c), indicating that, despite a high rate of variability of individual sites at the single-nucleotide level, the replicates were robust in terms of highly and lowly oxidized areas. To gain further insight into the nature of the DNA-modification distributions, we profiled oxidation across multiple levels of genomic resolution, including single nucleotides, genomic bins, functional elements, DNA strands and mtDNA, as well as analogously AP sites.

### DNA oxidation depends on local sequence contexts

To understand how patterns of DNA oxidation in cultured human cells may relate to mutational patterns from actual human cancer genomes, we determined the counts of oxidized guanines as a function of trinucleotide context (Fig. 2b) and compared this profile to single-base substitution (SBS) signatures from the Catalog of Somatic Mutations in Cancer (COSMIC)^33,34^ (Fig. 2c). Remarkably, the most similar SBS signature to the experimental data was SBS18, with cosine similarity in the range of 0.929–0.932 (Fig. 2c). SBS18 was established to have reactive oxygen species (ROS)-induced etiology according to experiments with human induced pluripotent stem cells exposed to potassium bromate^35^. Above the threshold of 0.85, two other putative ROS-related mutational signatures were similar to the experimental oxidation profile, namely, SBS30, associated with *NTHL1* deficiency^36^ (0.897–0.902 cosine similarity), and SBS36, associated with *MUTYH* deficiency^37,38^ (0.864–0.869 cosine similarity). Despite the overall high similarity between the damage profile and the mutational signature SBS18 (Fig. 2b), the mutation frequency is markedly higher than the oxidation frequency at certain trinucleotides, such as TGC, AGA and all CpG dimers CGN, which may indicate impaired repair in those contexts. On the contrary, there is an almost twofold drop in the frequency of mutations relative to oxidation in TGT and GGG trinucleotides, potentially reflecting preferential repair. Thus, the similarity between the trinucleotide-context-resolved levels of guanine oxidation and the mutational signature SBS18 found in human cancers is consistent with this signature originating from endogenous DNA oxidation.

**Extended Data Fig. 3:**
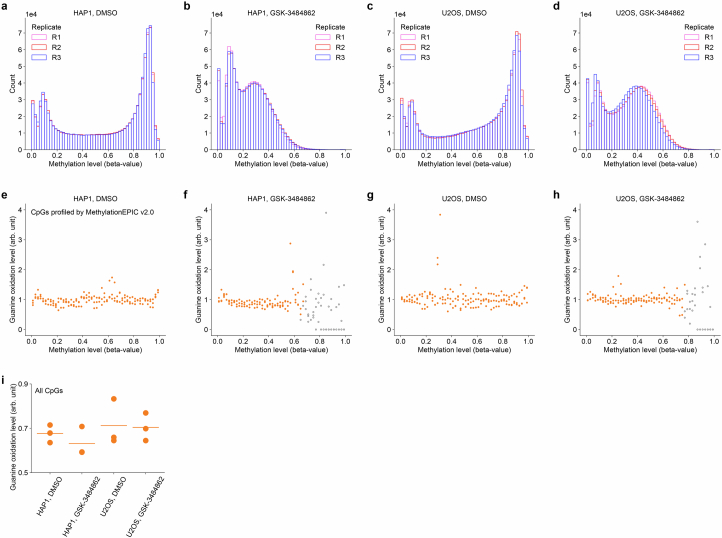
Average guanine oxidation level in CpG sites does not depend on cytosine methylation status. **a-d** Distribution of CpG site methylation levels profiled by Infinium MethylationEPIC v2.0 array (N = 920,026 CpG sites) in HAP1 cells exposed to DMSO (a), HAP1 cells exposed to DNA methyltransferase 1 inhibitor GSK-3484862 (b), U2OS cells exposed to DMSO (c), and U2OS cells exposed to GSK-3484862 (d) in n = 3 biological replicates. Binning with the step size 0.02. **e-h** Guanine oxidation level in CpG sites binned according to cytosine methylation level (the same as in **a-d**). At each bin of methylation level, respective values of oxidation in n = 3 biological replicates are shown. Gray empty markers: oxidation values in bins with ≤ 1,000 CpG sites (see respective panels **b** and **d**). **i** Guanine oxidation level in all nuclear-genome CpG sites in HAP1 and U2OS cells exposed to DMSO and GSK-3484862 (samples from **a-d**). Circular markers: n = 3 biological replicates per condition, horizontal dashes: means.

By relating the numbers of oxidized guanines in different sequence contexts to the abundance of these contexts in the human genome, we observed less oxidation of guanine when it is preceded by cytosine (CpG sites) compared to other nucleotides (Fig. 2d). As CpG sites are substrates of epigenetic cytosine methylation, we investigated the possible relationship of cytosine methylation and guanine oxidation by inducing hypomethylation and testing the impact on oxidation maps. For this, we exposed HAP1 and U2OS cells for 3 days to GSK-3484862, a DNA methyltransferase 1 (DNMT1) inhibitor^39^. Using the Infinium MethylationEPIC version 2.0 array, we confirmed a drastic hypomethylation such that the peak of highly methylated CpG sites (β values 0.8–1) found in vehicle (DMSO)-exposed cells is absent in GSK-3484862-exposed cells (Extended Data Fig. 3a–d). In contrast, the average oxidation level of guanines in the methylation-array-profiled CpG sites did not change in response to the inhibitor and was relatively constant amongst the CpG sites with varying methylation levels (Extended Data Fig. 3e–h). Moreover, there were no changes in guanine oxidation of all genomic CpG sites between DMSO and GSK-3484862 exposures (Extended Data Fig. 3i). Thus, in these cells, the oxidation at CpG sites, on average, does not appear to depend directly on cytosine methylation status.

Additionally, we searched for indications of at-a-distance oxidation because of electron–hole transport along the *π*-stacked base pairs^40^ but did not find expected preferential 5′-guanine oxidation in guanine doublets^41^ (Supplementary Fig. 6 and Supplementary Note 2)

### Drug-induced depurination is elevated in adenine dimers

Chemical and drug-induced DNA alkylation can promote base hydrolysis, leading to the formation of AP sites. To understand the genome-wide patterns of alkylating-agent-induced AP sites, we analyzed gDNA from U2OS cells exposed to the chemotherapeutic drug irofulven (6-hydroxymethylacylfulvene, HMAF)^27,42^ at 15 µM, which induced a fivefold increase in AP sites (Fig. 1f). Click-code-seq included ddNTP blocking of background strand breaks and incorporation of both prop-dGTP and prop-dATP to label AP sites. Consistent with expectations, we identified mostly adenine and guanine at the called genomic sites in both irofulven and vehicle conditions (Fig. 2e,f). In line with preferential alkylation at N^3^ adenine by irofulven^42^, the irofulven-induced depurination was observed more frequently at adenosines (Fig. 2e). In contrast, endogenous depurination (in vehicle-exposed cells) was mostly detected for guanosines (Fig. 2f), which agrees with snAP-seq^12^ but contradicts SSiNGLe-AP data^13^. In irofulven-exposed cells, we detected ~27–35 million unique AP sites, with only ~0.8–1% shared across replicates (Supplementary Fig. 7a,b); however, replicate genome-wide distributions were highly reproducible at 100-kbp binning (*r* = 0.98 for A and 0.96 for G) (Supplementary Fig. 7c), consistent with patterns observed for endogenous AP sites (Supplementary Fig. 7d–f). As a control for the detection of AP sites, we inserted uracils in a plasmid, converted them to AP sites by uracil-DNA glycosylase (UDG) and successfully called them in the expected locations (Extended Data Fig. 2b,e,f).

Regarding trinucleotide contexts of AP sites, in irofulven-exposed cells, these were preferentially present at adenine dimers (Fig. 2g, top, AAN). The profile of endogenous depurination was drastically different, with the highest modification levels at GGA and AGA (Fig. 2g, bottom). The DNA-modification pattern in irofulven-exposed cells was moderately similar (cosine similarity: 0.707–0.746) to the recently reported mutational signature of illudin S^43^, the natural-product precursor of irofulven (Supplementary Fig. 7g). Discrepancies were at TA dimers, suggesting a high mutagenicity of the relatively less frequent irofulven–DNA adduct in this sequence context, differences in sequence specificity of modification formation and repair between irofulven and illudin S or experimental differences. Comparing the DNA-modification pattern in irofulven-exposed cells to COSMIC mutational signatures, we uncovered a strong similarity with SBS40b (cosine similarity: 0.847–0.916) (Fig. 2g,h), an unknown etiology signature associated with clear cell renal cell carcinoma and biomarkers of impaired kidney function^44^. The pattern of endogenous AP sites was also highly similar to two mutational signatures of unknown etiology, namely, SBS39 (ref. ^45^) (cosine similarity: 0.924–0.945 (Fig. 2g, i) and SBS40a (ref. ^44^) (cosine similarity: 0.921–0.943) (Fig. 2i). Thus, SBS40b may be because of an exposure to an alkylating agent with similar biochemical properties to irofulven, and SBS39 and SBS40a may be because of endogenous depurination.

### DNA oxidation and AP sites correlate with chromatin state

**Fig. 3:**
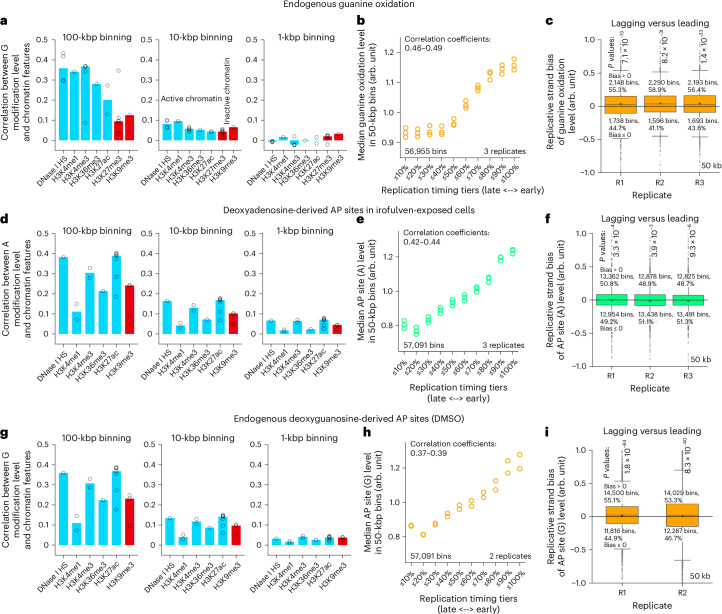
DNA oxidation and depurination levels correlate with chromatin marks and DNA replication timing. **a**,**d**,**g**, Spearman coefficient for chromatin marks versus endogenous guanine oxidation in HAP1 cells (**a**), irofulven-induced AP sites at adenines in U2OS cells (**d**) or endogenous AP sites at guanines in vehicle-exposed U2OS cells (**g**) with different genome binning. DNA modifications: for each replicate experiment, the signal was summed within bins, normalized by reference genome G (**a,g**) or A (**d**) and normalized by sequencing depth; *n* = 3 (**a**,**d**) or *n* = 2 (**g**) biological replicates were averaged per bin. Chromatin marks: each marker indicates a ChIP-seq mapping in a corresponding cell line under control conditions retrieved from ChIP-Atlas. The binning of ChIP-seq peaks is described in the Methods; a bar denotes the median correlation coefficient across available ChIP-seq experiments for a chromatin mark. **b**,**e**,**h**, Replication timing (Repli-seq log_2_ E/L RT) versus the level of endogenous guanine oxidation in HAP1 cells (**b**), irofulven-induced AP sites at adenines in U2OS cells (**e**) or endogenous AP sites at guanines in vehicle-exposed U2OS cells (**h**) in 50-kbp bins sorted in ten tiers of ascending Repli-seq signal (total number of bins indicated). Marker: median DNA-modification level across the bins of one tier in one biological replicate (number of replicates indicated). The range of Spearman coefficients across replicates is indicated. **c**,**f**,**i**, Replicative strand bias (lagging-strand template versus leading-strand template) of the level of endogenous guanine oxidation in HAP1 cells (**c**), irofulven-induced AP sites at adenines in U2OS cells (**f**) or endogenous AP sites at guanines in vehicle-exposed U2OS cells (**i**) in 50-kbp bins in multiple replicates. The number and fraction of bins with positive and nonpositive bias values and two-tailed Wilcoxon signed-rank test *P* values are provided. Boxes: interquartile ranges; internal horizontal lines: medians; stars: means; whiskers: extending to the furthest data point within 1.5× the interquartile range; data points beyond: small markers.

Moving to large genomic bins, we noticed varying guanine oxidation levels along chromosomes even after correcting for the guanine count per bin (1st percentile: 0.68–0.73, 99th percentile: 2.15–2.22 at 100 kbp) (Supplementary Fig. 8a). To test whether this variation could be explained by chromatin state, we related guanine oxidation levels with chromatin features, derived from the ChIP-Atlas^46^ data for HAP1 cells. Here, we found that, at 100-kbp resolution, guanine oxidation levels positively correlated with euchromatin features, namely, DNase I hypersensitivity sites (transcriptional activity), H3K4me1 (active enhancers), H3K4me3 (active gene promoters), H3K36me3 (active gene bodies) and H3K27ac (active promoters and enhancers) (Fig. 3a, 100 kbp). There also was a positive, albeit much weaker, correlation with heterochromatic marks, namely, H3K27me3 and H3K9me3 (Fig. 3a, 100 kbp). As the average ChIP-seq peak size for the chromatin marks is in the range of 118–1,023 bp (HAP1), we more finely binned the ChIP-seq and guanine oxidation data and observed that the correlations were not preserved at higher resolutions, falling to around zero at 1-kbp binning (Fig. 3a). We made similar observations after matching irofulven-induced (Fig. 3d and Supplementary Fig. 8b) or endogenous (Fig. 3g and Supplementary Fig. 8c) AP sites with chromatin features in the U2OS-cell genome at increasing resolutions. Thus, while the presence of a euchromatin or heterochromatin mark in a genomic bin is moderately to weakly associated with higher DNA-modification levels at 100-kbp resolution, the distributions of guanine oxidation and AP sites do not seem to be primarily shaped by chromatin state locally (that is, at 1-kbp resolution), where other genomic features could have more influence.

Integration of replication timing (Repli-seq) data^47,48^ revealed the enrichment of DNA modifications in early replicating regions (Fig. 3b,e,h), and a weak but consistent strand bias toward the lagging-strand template for endogenous oxidation and depurination (Fig. 3c,f,i and Supplementary Note 3).

### Guanine oxidation has a transcriptional strand bias

**Fig. 4:**
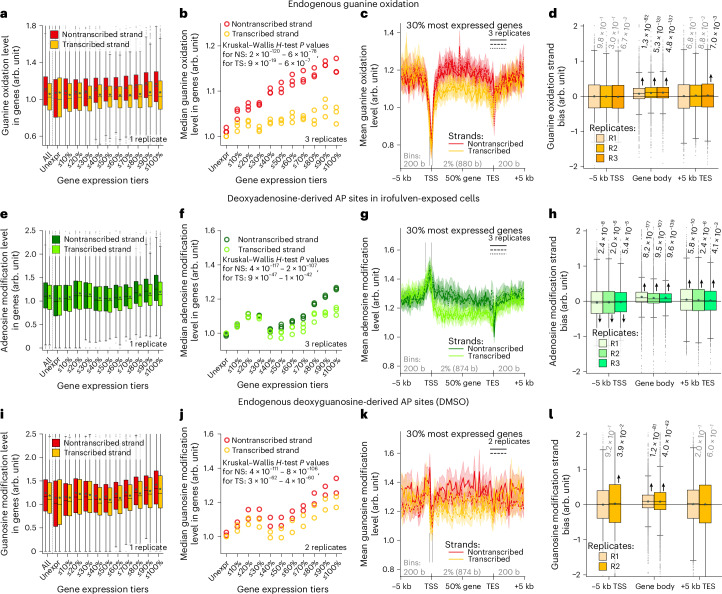
Oxidized guanines and AP sites have transcriptional strand biases. **a**–**d**, Endogenous guanine oxidation around gene bodies in HAP1 cells. **e**–**h**, Irofulven-induced AP sites at adenines around gene bodies in U2OS cells. **i**–**l**, Endogenous (DMSO) AP sites at guanines around gene bodies in U2OS cells. **a**,**e**,**i**, DNA-modification level in gene-body strands in protein-coding genes from one biological replicate versus gene expression level. **b**,**f**,**j**, Median DNA-modification level in gene-body strands in protein-coding genes in multiple biological replicates versus gene expression level. **c**,**g**,**k**, Strand-specific profile of the mean DNA-modification level and its 95% confidence interval throughout the gene body and its upstream and downstream regions in highly expressed genes (**c**, *n* = 3,994; **g**,**k**, *n* = 4,425); solid, dashed and dotted curves with shades: different biological replicates; genes are aligned at the TSS and TES; vertical dashes demark TSS-adjacent regions analyzed in Extended Data Fig. 8. **d**,**h**,**l**, Strand bias (difference) of DNA-modification levels between the nontranscribed and transcribed strands within the gene body and adjacent upstream and downstream 5-kbp regions for the 30% most expressed genes (**d**, *n* = 3,994; **h**,**l**, *n* = 4,425) in multiple replicates. *P* values of a two-tailed Wilcoxon signed-rank test are provided. The arrow shows the shift of the median from 0 if *P* < 0.05. In **a**,**d**,**e**,**h**,**i**,**l**, boxes are interquartile ranges, internal horizontal lines are medians, stars are means, whiskers extend to the furthest data point within 1.5× the interquartile range and data points beyond are shown as small markers. The number of genes per tier and number of genes beyond the minimal and maximal *y* axis values are indicated in the Methods. In **b**,**f**,**j**, we provide the min–max ranges of *P* values of the Kruskal–Wallis *H*-test performed for either strand across all replicates. DNA-modification level normalization is described in the Methods.

**Extended Data Fig. 4:**
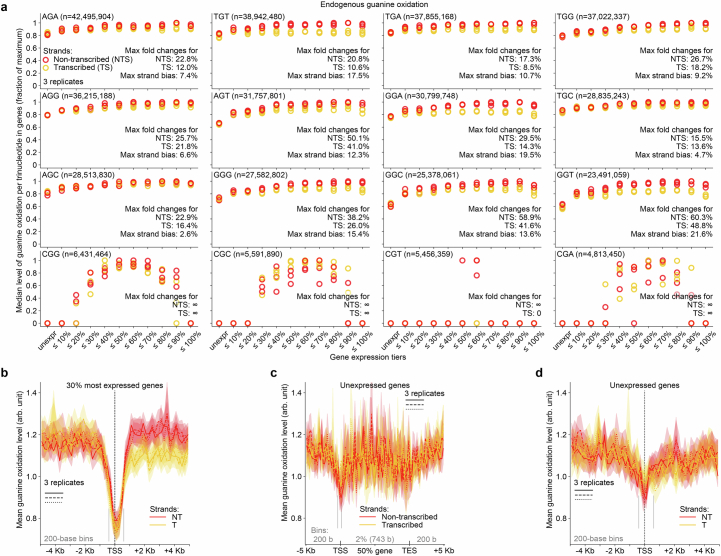
Oxidized guanines accumulate in gene bodies with increasing gene expression and show a strand bias. **a** Median level of endogenous guanine oxidation in protein-coding genes considered separately for individual trinucleotide contexts and strands versus gene expression in HAP1 cells. The level of trinucleotide-specific guanine oxidation is normalized by the count of the respective trinucleotide within each protein-coding gene in the reference genome. For each replicate, the level of guanine oxidation is further normalized by the maximal value among the medians of gene-expression tiers and strands (for each sample, the maximal – among all tiers and strands – trinucleotide-specific value is set to 1; see normalization formulas in Methods). The trinucleotides are sorted according to the total occurrence within the protein-coding genes in the reference genome, with respective numbers indicated in parentheses. Maximal fold change for a strand: we averaged the replicate values at each tier, related these averages to the average of the unexpressed genes and indicated the maximal percentage of the change. Maximal strand bias: for every tier starting from ≤40%, we related the averages of the replicate medians between the non-transcribed and transcribed strands and indicated the maximal percentage of the change. **b-d** Strand-specific profile of the mean guanine-oxidation level and its 95% c.i. throughout the gene body and its upstream and downstream regions in highly expressed (**b**: n = 3,994) and unexpressed (**c-d**: n = 3,428 genes); solid, dashed, and dotted curves with shades: different biological replicates; vertical dashes demark TSS-adjacent regions analyzed in Extended Data Fig. 8a-b; genes are aligned only at TSS (**b,d**) or at both TSS and TES (**c**). Arb. unit: Methods describe guanine-oxidation level normalization. NT: non-transcribed, T: transcribed. TTS and TES: transcription start and end sites. Kb: kilobase; b: base.

Having observed DNA-modification association with active chromatin, we focused the analysis on gene regions. We measured guanine oxidation levels in genes between transcription start and end sites (TSSs and TESs), separately in the transcribed and nontranscribed strands, and related these levels to gene expression. To account for different guanine abundances in the strands, we computed guanine oxidation level as the ratio of number of mapped oxidized guanines to all guanines in the respective gene and strand. To compare guanine oxidation levels amongst replicate samples independent of sequencing depth, we normalized all genomic-feature-specific guanine oxidation levels by the median guanine oxidation level in the transcribed (antisense) strands of unexpressed genes (Fig. 4a; median of the second yellow box set to 1 by this normalization). We found no overall strand bias for occurrence of guanine oxidation in the group of unexpressed genes (Fig. 4a, unexpr). However, with increasing gene expression, guanine oxidation levels continuously increased, especially in the nontranscribed versus transcribed strand (Fig. 4a). This gene-expression-dependent strand bias of guanine oxidation was robust across biological replicates, with median levels increasing maximally by 14.6% for the nontranscribed strand but only by 6.3% for the transcribed strand, both relative to the unexpressed gene tier (Fig. 4b). Excluding a potential confounding factor of varying sequence composition across gene expression tiers, we observed the increasing guanine oxidation level and widening strand bias with gene expression also when focusing on individual trinucleotides within genes (Supplementary Note 4 and Extended Data Fig. 4a).

For highly expressed genes, we observed a strand bias of guanine oxidation only throughout the gene body but not upstream of the TSS or downstream of the TES (Fig. 4c and Extended Data Fig. 4b–d), which was further supported by a Wilcoxon signed-rank test (Fig. 4d).

### AP sites have a transcriptional strand bias

**Extended Data Fig. 5:**
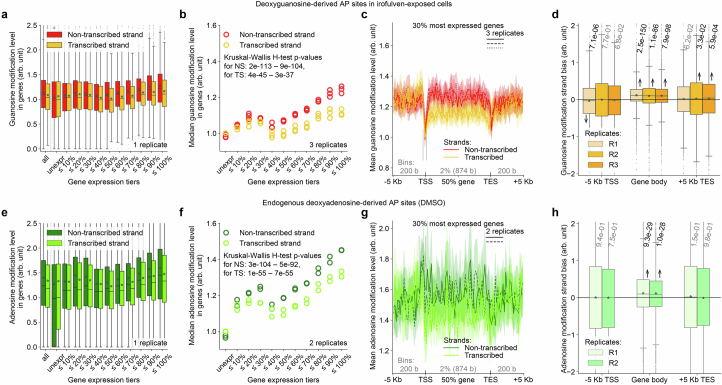
AP sites accumulate in gene bodies with increasing gene expression and show a strand bias. **a-d** Guanosine-derived AP sites in irofulven-exposed U2OS cells throughout gene bodies and adjacent regions. **e-h** Endogenous adenosine-derived AP sites in vehicle-(DMSO)-exposed U2OS cells throughout gene bodies and adjacent regions. The plots are built analogously to Fig. 4e-l.

To explore how transcription may relate with irofulven-induced and endogenous AP sites, we analyzed their gene expression dependency, separately for adenosines and guanosines, focusing on one biological replicate (Fig. 4e,i and Extended Data Fig. 5a,e) and showing medians of multiple replicates (Fig. 4f,j and Extended Data Fig. 5b,f). For irofulven-induced AP sites (Fig. 4f and Extended Data Fig. 5b), we observed 27.3% (at adenosines) and 26.3% (at guanosines) elevations of median DNA modification level for the nontranscribed strand in the most expressed genes (≤100%) compared to unexpressed ones. For the transcribed strand, the maximal elevation of median DNA modification was 13.1% for adenosines and 11.4% for guanosines (Fig. 4f and Extended Data Fig. 5b), creating a pronounced strand bias in DNA modification throughout the second half of gene expression tiers (maximally 11.5% for both A and G), which is in line with irofulven-induced adducts being known transcription-coupled nucleotide excision repair (TC-NER) substrates^49,50^.

**Extended Data Fig. 6:**
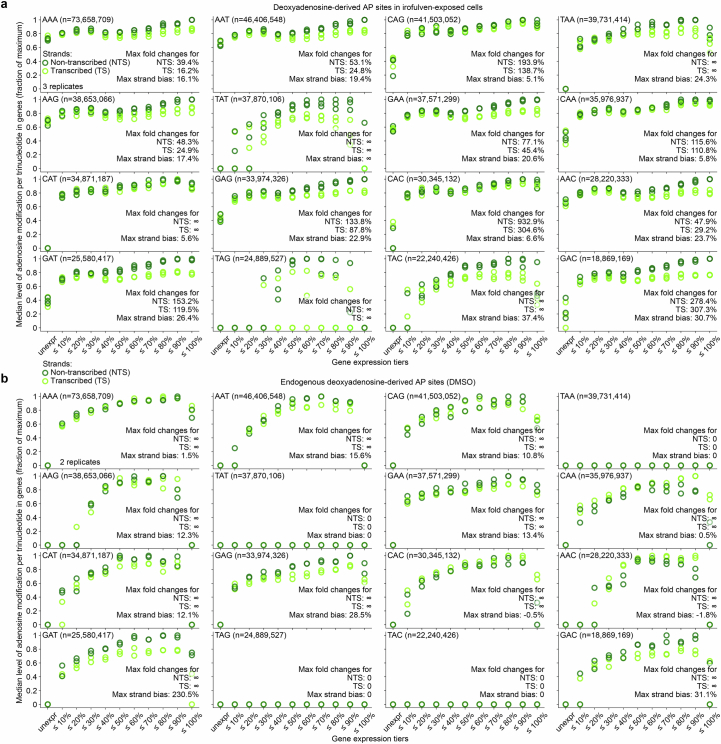
Trinucleotide-context analysis: deoxyadenosine-derived AP sites accumulate in gene bodies with increasing gene expression and show a strand bias. **a-b** Median level of deoxyadenosine-derived AP sites in irofulven-exposed (**a**) or vehicle-(DMSO)-exposed (**b**) U2OS cells in protein-coding genes considered separately for individual trinucleotide contexts and strands versus gene expression in unexposed U2OS cells. The plot is built analogously to Extended Data Fig. 4a.

**Extended Data Fig. 7:**
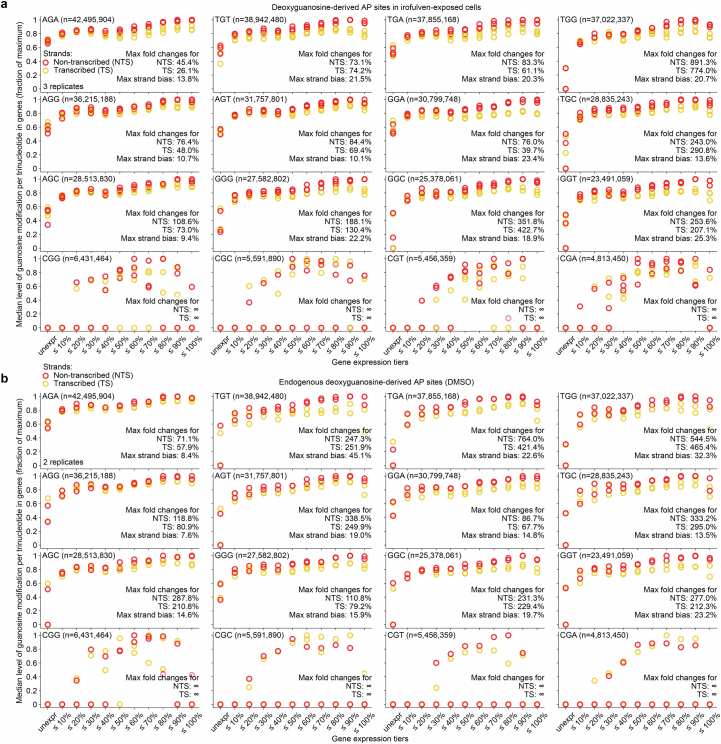
Trinucleotide-context analysis: deoxyguanosine-derived AP sites accumulate in gene bodies with increasing gene expression and show a strand bias. **a-b** Median level of deoxyguanosine-derived AP sites in irofulven-exposed (**a**) or vehicle-(DMSO)-exposed (**b**) U2OS cells in protein-coding genes considered separately for individual trinucleotide contexts and strands versus gene expression in unexposed U2OS cells. The plot is built analogously to Extended Data Fig. 4a.

We identified that irofulven-induced AP sites derived from adenosine alkylation prevail in the nontranscribed strand throughout the gene body and downstream of TES (Fig. 4g), as reproduced in three replicates (Fig. 4h); however, the strand bias is swapped upstream of the TSS such that the AP sites occur more frequently in the transcribed strand (Fig. 4g,h). A significant strand bias of irofulven-induced AP sites derived from guanosine was found only throughout the gene body (Extended Data Fig. 5c,d). As for endogenous depurination, we also observed elevating AP site levels (maximally 49.6% for A, 27.7% for G) and a widening strand bias with increasing gene expression (maximally 10% for A, 8% for G), only within the gene body, for both guanosines (Fig. 4i–l) and adenosines (Extended Data Fig. 5e–h). We observed that DNA-modification burdens increased with gene expression for almost all trinucleotides in both irofulven-induced and endogenous depurination, yet trinucleotides varied in maximal fold changes and strand biases of AP site levels (Extended Data Figs. 6 and 7). Particularly for adenosine-derived AP sites, gene-expression-dependent patterns were notably different between vehicle-exposed and irofulven-exposed samples, supporting that the endogenous adenosine-derived AP sites do not confound the mapping of irofulven-induced ones (Supplementary Note 5).

**Extended Data Fig. 8:**
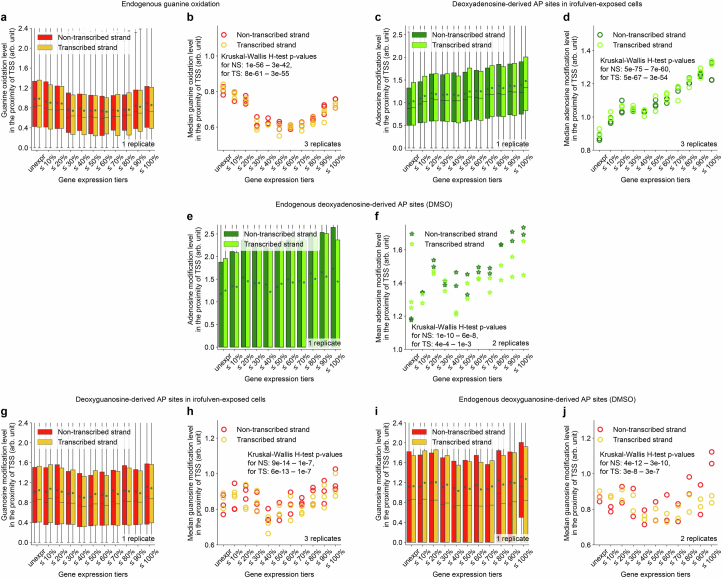
DNA-modification level around the transcription start site in each strand in protein-coding genes versus gene expression level. **a-b** Endogenous guanine oxidation in HAP1 cells between -400 and +600 b relative to TSS. **c-f** Deoxyadenosine-derived AP sites in irofulven-exposed (**c-d**) or vehicle-(DMSO)-exposed (**e-f**) U2OS cells between -1000 and 0 b relative to TSS. **g-j** Guanosine-derived AP sites in irofulven-exposed (**g-h**) or vehicle-(DMSO)-exposed (**i-j**) U2OS cells between -400 and +600 b relative to TSS. Plots are built analogously to Fig. 4a-b. In panels **a**,**c**,**e**,**g**,**i**, boxes are interquartile ranges, internal horizontal lines are medians, stars are means, whiskers extend to the furthest datapoint within 1.5x interquartile range, datapoints beyond are shown as small markers; number of genes per tier and number of genes beyond the minimal and maximal y-axis values are indicated in Methods. In panels **b**,**d**,**f**,**h**,**j**, we provide the min-max ranges of p-values of the Kruskal–Wallis H test performed for either strand across all replicates. Arb. unit: Methods describe DNA-modification level normalization. TTS: transcription start site. b: base.

Guanine oxidation and AP sites demonstrate divergent patterns in the proximity of the TSS: a sharp drop in guanine oxidation (Fig. 4c) (which has been disputed in the literature with both depletion^14,18,20^ and enrichment^15,21,22^ observations) and a spike in irofulven-induced adenosine depurination (Fig. 4g). We further found that burdens of DNA oxidation and alkylation-induced and endogenous depurination in promoters vary with increasing gene expression (Extended Data Fig. 8 and Supplementary Note 6).

### Strand bias of endogenous oxidation and AP sites in mtDNA

**Fig. 5:**
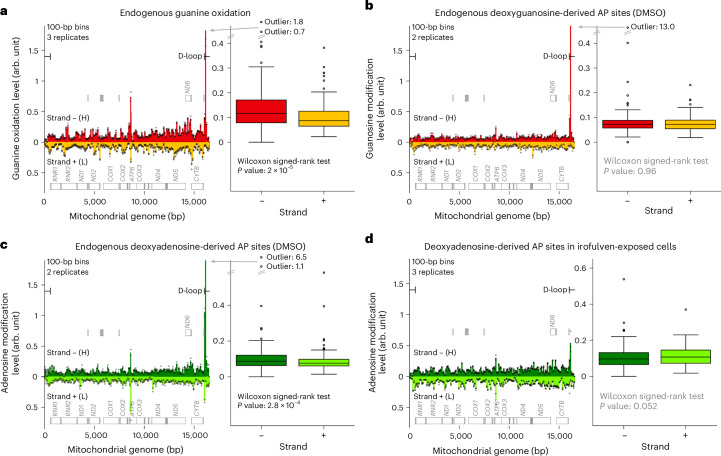
Endogenous guanine oxidation and adenosine depurination are more frequent in the − (heavy) strand of mtDNA, while endogenous guanosine depurination and drug-induced depurination do not have an overall strand bias. **a**–**d**, Left, the strand-specific profile of endogenous guanine oxidation in HAP1 cells (**a**), endogenous guanosine (**b**) and adenosine (**c**) depurination in U2OS cells (DMSO vehicle) and adenosine depurination in irofulven-exposed U2OS cells (**d**) throughout mtDNA. We aggregated DNA modifications in consecutive 100-base bins for each strand and normalized these bin-specific and strand-specific counts by the total replicate-specific DNA-modification count in mtDNA and further by the bin-specific and strand-specific target-base count from the reference genome. Markers: biological replicates (number specified); bars: means of the replicate values. The locations of 37 genes are provided as gray boxes along their sense strands. H, heavy; L, light. Right, bin-specific mean values from the left panel (bars) aggregated within each strand. Wilcoxon signed-rank test: *n* = 166 pairs of strand-specific values (means across the biological replicates, number specified in the left panel), two-sided alternative. Boxes are interquartile ranges, internal horizontal lines are medians, whiskers extend to the furthest data point within 1.5× the interquartile range, data points beyond are shown as markers and the values of the most extreme outliers are provided.

**Extended Data Fig. 9:**
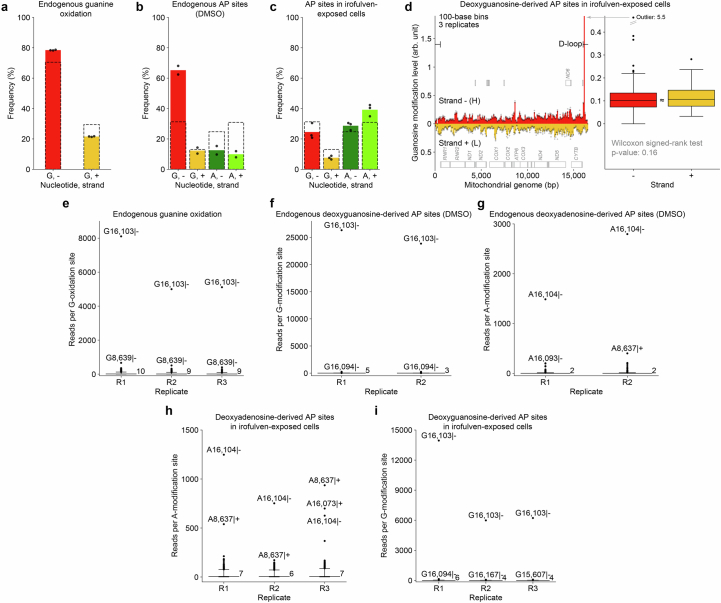
The distribution of DNA modifications in mtDNA. **a-c** The distribution of endogenous DNA oxidation in HAP1 cells (**a**), AP sites in vehicle-(DMSO)-exposed U2OS cells (**b**) and AP sites in irofulven-exposed U2OS cells (**c**) throughout the mtDNA strands and affected nucleosides; markers, replicates; filled bars, means. Dashed bars, the distribution of respective nucleotides according to the reference genome. **d** Left panel: The strand-specific profile of guanosine depurination in irofulven-exposed U2OS cells throughout mtDNA. Right panel: bin-specific mean values (bars) from the left panel aggregated within each strand. Wilcoxon signed-rank test: n = 166 pairs of strand-specific values, two-sided alternative. The plots are built analogously to Fig. 5. **e-i** The distribution of the number of unique reads (with individual RICs) supporting a DNA modification site in mtDNA across the replicate experiments. Distribution plot from the bottom to the top: short horizontal line, 1^st^ percentile; long horizontal line, median, the value is provided; short horizontal line, 99^th^ percentile; markers, values bigger than the 99^th^ percentile. The coordinates of the sites with highest read coverages are provided.

Lastly, we focused on mtDNA, which has been largely overlooked by previous DNA-modification mapping methods, although mtDNA is particularly vulnerable to modifications because of its proximity to the electron transport chain, a major source of ROS. The heavy (−) and light (+) strands of mtDNA differ in guanine content (Extended Data Fig. 9a, dashed bars) and, interestingly, we observed a higher-than-proportional prevalence of oxidized guanines in the − strand (Extended Data Fig. 9a, filled versus dashed bars). Concerning the propensity for oxidized guanine to exist throughout mtDNA, when we aggregated data in 100-base bins, most in the − strand had higher guanine oxidation levels compared to their counterparts in the + strand (Fig. 5a).

Through a similar analysis, endogenous guanosine-derived AP sites showed no significant overall strand bias (Fig. 5b), while the less abundant (Extended Data Fig. 9b) endogenous adenosine-derived AP sites mapped in the same samples were significantly more frequent in the − strand than in the + strand throughout mtDNA (Fig. 5c), consistent with recent findings in mouse tissues^13^. Upon irofulven exposure, most AP sites originated from adenosine (Extended Data Fig. 9c), with no significant strand bias for both adenosine-derived and guanosine-derived AP sites (Fig. 5d and Extended Data Fig. 9d).

DNA modifications had two prominent peaks, namely, in the gene *ATP6* encoding ATP synthase membrane subunit 6 and in the D-loop region (Fig. 5 and Extended Data Fig. 9d). The D-loop peak was driven mainly by single-nucleotide signals, G16103 and A16104, near the 3′ end of 7S DNA, suggesting a role in regulating 7S DNA synthesis termination and mtDNA replication^51,52^ (Extended Data Fig. 9e–i and Supplementary Note 7).

## Discussion

We uncovered new insights into the uneven distribution of endogenous DNA oxidation and AP sites in human gDNA and generated the first genome-wide map of DNA modifications induced by irofulven, an emerging precision-oncology drug^53,54^. This was enabled by two complementary methods: click-fluoro-quant, a rapid fluorescence-based assay for quantifying oxidized guanines, AP sites and DNA breaks, and click-code-seq, a single-nucleotide-level DNA-modification sequencing method. The endogenous guanine oxidation profile in human cells closely matched the ROS-associated mutational signature SBS18 observed in cancers. CpG dimers showed the lowest endogenous guanine oxidation among guanine-containing dinucleotides, yet it proved not to depend on cytosine methylation status. In irofulven-exposed cells, AP sites were enriched at adenine dimers and resembled the kidney-cancer-associated mutational signature SBS40b. Moreover, we found a transcription-linked strand bias: transcribed strands accumulated fewer oxidized guanines and AP sites than nontranscribed strands, with the bias increasing with gene expression. Additionally, mtDNA strands showed asymmetric endogenous guanine oxidation and adenosine depurination.

We addressed several fundamental factors that reduce the specificity of DNA-modification sequencing. First, target-versus-background differentiation is a common issue in methods such as SSiNGLe-AP^13^ (AP sites versus breaks), CLAPS-seq^14^ (8-oxoG versus polymerase stalling modifications such as AP sites) and 250-bp-resolution AP-seq^20^ (8-oxoG versus AP sites). As click-code-seq tags 3′-OH groups, background DNA breaks and AP sites can confound the mapping of oxidized guanines and AP sites recognized through glycosylase and endonuclease treatment. To address this, we used click-fluoro-quant data to evaluate library preparation efficiency and blocked background modifications, as performed in SSiNGLe-AP^13^. Second, we postponed sonication until after labeling target modifications to avoid unintended DNA modifications leading to false positives^23^. Methods such as OG-seq^15^ and OxiDIP-seq^21^ with >100-bp resolution involve DNA sonication before labeling 8-oxoG, although antioxidants were used. In AP-site-specific SSiNGLe-AP^13^ and thymidine-glycol-specific DPC-seq^55^, DNA was fragmented enzymatically rather than through sonication. By performing sonication after labeling and adding antioxidants during handling until labeling, we protected DNA from oxidative artifacts. Third, most DNA-modification sequencing methods rely on PCR amplification of enriched DNA fragments, which can introduce unspecific products because of spurious priming and obscure whether site is modified in one or multiple cells. We devised an adaptor, MoDIS, comprising a VC to filter out unspecific PCR products and an RIC to assess modification frequency across cells. Accordingly, we found that 14–21% of reads were unspecific (Supplementary Figs. 3c and 4, reads without VC), indicating that spurious amplification may substantially affect data from methods lacking such controls. Furthermore, click ligation of MoDIS may outperform enzymatic ligation^56^ used in other sequencing methods and the resulting triazole linkage does not stall polymerase^18,57^. The current protocol combines elements of both the original version^18^ and click-code-seq version 2.0 (ref. ^25^) (Supplementary Discussion).

There is substantial evidence that transcription compromises genome integrity^58–60^. In line with this notion, we found that, in human cells, the levels of both endogenous guanine oxidation (Fig. 4a,b) and irofulven-induced (Fig. 4e,f) and endogenous depurination (Fig. 4i–j) are elevated in gene bodies with increasing gene expression, which aligns with a similar recent observation of endogenous AP sites in mouse tissues^13^. These findings suggest that DNA becomes more vulnerable to damaging agents, such as ROS and irofulven, and hydrolysis during transcription. This vulnerability may be because of the strand separation and single-strandedness of DNA during transcription, particularly in transcription bubbles, potentially in cotranscriptional R-loops^61^ and in negative supercoils behind RNA polymerases^59,60^. Single-stranded DNA provides greater accessibility of nucleobases to damaging agents, as observed by higher rates of spontaneous depurination^24^ and spontaneous deamination^62^ in single-stranded versus double-stranded DNA. Transcription bubbles or R-loops could explain the observed gene-expression-dependent strand biases (that is, more DNA modifications in the nontranscribed strand of the gene body; Fig. 4d,h,l) as the transcribed strand is involved in DNA–RNA hybrid duplexes^61^, leaving the nontranscribed strand exposed. In studies with mutation reporters in bacteria^63–65^ and yeast^66^, this mechanism for the emergence of transcription-dependent strand bias was suggested for deamination of cytosine to uracil, guanine oxidation^64^ and alkylation by methylmethane sulfonate^65^. Transcription-associated negative supercoiling, which can make both DNA strands more exposed^59,60^, may explain why the transcribed strand also shows a slight increase of all mapped DNA modifications with rising gene expression (Fig. 4b,f,j). Further supporting the idea that single-stranded DNA is more susceptible to endogenous guanine oxidation and depurination, we observed a weak replicative strand bias of these modifications toward the lagging-strand template (Fig. 3c,i), a known source of single-stranded DNA in the cell^67^, although sequence motif specificity of these DNA modifications could potentially correlate with the sequence bias for replication. In contrast, irofulven-induced AP sites did not exhibit a consistent replicative strand bias (Fig. 3f), suggesting that DNA single-strandedness may not be a major determinant of irofulven-induced DNA modification patterns; however, a replication-specific mechanism cannot be ruled out.

Additionally, the observed transcription-linked strand biases of DNA modifications could be attributed to transcription-coupled repair, particularly TC-NER, which is initiated by RNA polymerase stalling at DNA modification and operates in the transcribed strand. Such TC-NER-attributed strand biases have been observed for bulky DNA modifications such as cyclobutane pyrimidine dimers^16^ and benzo[a]pyrene-induced adducts^68^ mapped across the genome in human cells. The depletion of irofulven-induced AP sites in the transcribed strand agrees with the fact that irofulven–DNA or, in general, acylfulvene–DNA adducts are known TC-NER substrates^42,50,69,70^. Under this model, the irofulven–DNA adducts are formed in double-stranded DNA at the N^3^ position of adenine in minor groove^42^; then, TC-NER repairs the alkylated transcribed strands and ignores irofulven–DNA adducts in the nontranscribed strand, which in turn spontaneously depurinate intracellularly or during DNA-sample incubation, manifesting in the observed transcriptional strand bias of AP sites in irofulven-exposed cells (Fig. 4g,h). Similar strand biases for endogenous guanine oxidation and AP sites may suggest that these modifications are also removed by transcription-coupled repair. Supporting this, AP sites have been shown to block transcription^71^ and are processed by TC-NER as shown in yeast^72^. Although 8-oxoG does not block transcription directly^73–75^, there is evidence that TC-NER can still contribute to the removal of 8-oxoG, with the CSB protein of TC-NER facilitating BER progression at expressed genes^76^ and with BER intermediates (AP sites and breaks) blocking transcription and eventually being resolved by TC-NER^75,77,78^. In contrast to 8-oxoG, other guanine oxidation products, namely, guanidinohydantoin (Gh) and spiroiminodihydantoin (Sp), cause strong RNA polymerase II stalling and may trigger TC-NER^79^. As FPG used by click-code-seq can recognize and excise Gh and Sp^80^, their TC-NER processing may contribute to the observed transcription bias of guanine oxidation. Interestingly, endogenous single-strand breaks also show a gene-expression-dependent strand bias; however, contrary to oxidation and depurination profiled in this work and most existing evidence for DNA modifications, slightly more breaks are formed in the transcribed strand^7^. Further studies with DNA-modification sequencing, especially in systems deficient in specific DNA repair pathways, are needed to disentangle the respective contributions of DNA-modification formation and repair in shaping transcription-linked DNA modification patterns.

Promoter regions also show complex DNA modification patterns. Notably, the strand bias of irofulven-induced adenosine-derived AP sites was inverted for at least 5 kbp upstream of the TSS, with the transcribed strand (from the gene-body perspective) accumulating more DNA modifications (Fig. 4g,h). This mirrors findings with trabectedin, another TC-NER-specific chemotherapeutic drug^7^, suggesting that TC-NER activity extends kilobases upstream, likely connected to divergent transcription^81^. Interestingly, echoing trabectedin data^7^, there is a 1-kbp region immediately upstream of the TSS that appears to be a TC-NER blind spot, where the level of irofulven-induced adenosine-derived AP sites peaks without a strand bias (Fig. 4g) and increases with gene expression (Extended Data Fig. 8c,d), which may promote drug toxicity in a TC-NER-independent manner.

We found that endogenous guanine oxidation is lowest at CpG sites compared to other dinucleotides with guanine in the 3′ position. Lower levels of guanine oxidation at CpG sites may be less disruptive to epigenetic methylation patterns on 5′-flanking cytosines, as 8-oxoG can both inhibit DNMTs^82–84^ and promote active demethylation through TET1 enzyme recruitment by 8-oxoguanine DNA glycosylase (OGG1)^85^. However, when we tested the hypothesis that cytosine methylation might reduce susceptibility to oxidation or enhance repair, there was no impact of inducing hypomethylation on guanine oxidation at CpG sites. Prior research related to this question involved analysis of 8-oxoG in oligonucleotides and provided conflict as to whether methylation interferes with propensity for excision of adjacent 8-oxoG by OGG1^86^ versus guanine oxidation by oxidants^87^. Further investigation is required to uncover the molecular basis for lower levels of endogenous guanine oxidation observed at CpG sites in this context and the interplay between DNA methylation and oxidation.

In mtDNA, endogenous guanine oxidation and adenosine depurination were more common throughout the − strand, which is mostly the transcribed strand (Fig. 5a,c). This contrasts patterns in nuclear DNA, where there is less guanine oxidation and depurination in the transcribed strand. This difference likely reflects the strand displacement model of mtDNA^52^, where − strand synthesis begins much earlier than that of the + strand, leaving the old − strand unpaired and exposed. However, there is no such strand asymmetry for endogenous guanosine depurination (Fig. 5b) nor irofulven-induced depurination (Fig. 5d), possibly because of opposing effects of replication (exposing the − strand) and transcription (exposing the nontranscribed + strand for most genes). For endogenous guanine oxidation and adenosine depurination, the replication factor may dominate, leading to the observed strand asymmetries. These strand imbalances in DNA modifications may underlie mechanisms driving mtDNA instability^88^.

The single-nucleotide resolution of click-code-seq allowed us to correlate DNA-modification frequencies in trinucleotide contexts with cancer mutational signatures, providing clues to their origin. The pattern of endogenous guanine oxidation closely matched COSMIC signature SBS18 (Fig. 2c), associated with ROS, supporting this annotation. This adds to prior examples of forecasting human mutational signatures with DNA-modification signatures, as observed for human lung cancers versus cultured lung cells exposed to a tobacco carcinogen^68^ and cancers in persons previously treated with the glioblastoma drug temozolomide versus cultured glioblastoma cells exposed to temozolomide^17^. Here, we uncovered a close match between the AP site pattern in irofulven-exposed cells and SBS40b, a kidney cancer signature of unknown mutagenic exposure varying across countries^44^, indicating a possible link to an irofulven-like alkylating agent. This parallels the mutational signature of aristolochic-acid exposure and kidney disease^89^, particularly given that DNA adducts of aristolochic acid, like irofulven’s, have biological effects associated with TC-NER^90^.

## Methods

Unless otherwise stated, chemicals were acquired from Sigma-Aldrich, molecular biology reagents were acquired from New England Biolabs (NEB) and cell culture reagents were acquired from Thermo Fisher. Oligonucleotide DNA was synthesized and purified using PAGE or reverse-phase high-performance liquid chromatography (HPLC) by Eurogentec. Adaptors and primers for click-code-seq were synthesized and HPLC-purified by Eurogentec. Solutions were prepared in MilliQ-purified H_2_O. Buffer EB (Qiagen, 19086) contained 10 mM Tris-Cl pH 8.5. Cell lines were authenticated by Microsynth.

### Cell culture and exposure to KBrO_3_, irofulven, GSK-3484862 and UVA

HAP1 cells (Horizon Discovery, C631, RRID:CVCL_Y019) were cultivated in Iscove’s modified Dulbecco’s medium (Thermo Fisher, 12440053) supplemented with 10% FBS (Thermo Fisher, A5256701) and 1% penicillin–streptomycin (Thermo Fisher, 15140122) at 37 °C, 5% CO_2_ and 3% O_2_. U2OS cells (RRID:CVCL_0042, M. Rubin’s lab, Department for BioMedical Research, University of Bern) were cultivated in McCoy’s general cell culture medium, modified with high glucose, L-glutamate, Bacto peptone and phenol red, lacking sodium pyruvate and HEPES (Thermo Fisher 16600082) and supplemented with 10% FBS (Thermo Fisher, 10270106) and 1% penicillin–streptomycin (Thermo Fisher, 15140122) at 37 °C, 5% CO_2_ and, in the case of DNMT1 inhibition, 3% O_2_ (see below). BJ-5ta cells (American Type Culture Collection, CRL-4001) were cultured at 37 °C, 5% CO_2_, 3% O_2_ in a medium composed of four parts DMEM (Thermo Fisher, 31966-021), one part Medium 199 (Thermo Fisher, 22340-020), 10% FBS (Thermo Fisher, A5256701) and 0.01 mg ml^−1^ hygromycin B (Sigma-Aldrich, H3274).

For KBrO_3_ treatment, medium was removed and cells were washed with 10 ml of Dulbecco’s PBS (Thermo Fisher, J67670.AP). KBrO_3_ (Sigma-Aldrich, 309087) was dissolved in 10 ml of medium for a final concentration of 50 mM and added to the cells, followed by a 30-min incubation at 37 °C and harvesting. For the exposure to irofulven (provided by MGI Pharma, currently part of Eisai), medium was prepared by adding HMAF (50 mM stock, dissolved in DMSO) to 10 ml of medium to a final concentration of 0.8–15 μM. A control medium was prepared by adding DMSO (Sigma-Aldrich, D8418) in the same volume as the treatment to 10 ml of medium. The treatment and control media were filtered with a sterile syringe filter (0.22 μm) and added to the cells followed by 4-h incubation at 37 °C. For UVA treatment, medium was replaced with serum-free medium. Dishes were covered with soda-lime glass lids to filter out potential UVB contamination from the UV lamp. Cells were irradiated in a UV irradiation chamber (Opsytec Dr. Gröbel, BS-02) with an intensity of 8 mW cm^−2^ for a final dose of 10 J cm^−2^ (around 18 min) controlled by a dosimeter. For DNMT1 inhibition experiment with HAP1 and U2OS cells, 10 ml of of cell culture medium was supplemented with 4 μl of GSK-3484862 (MedChemExpress, HY-135146; 10 mM, dissolved in DMSO; final concentration: 4 μM). Control medium was prepared by adding 4 μl of DMSO to 10 ml of cell culture medium. The treatment and control media were filtered with a sterile syringe filter (0.22 μm) and added to the cells, followed by a 72-h incubation at 37 °C, 5% CO_2_ and 3% O_2_, with the treatment and control media being refreshed after every 24 h.

### gDNA extraction and sonication

Cells were harvested by removing the medium and washing once with 10 ml of DPBS (Thermo Fisher, J67670.AP), followed by the addition of 1 ml of 0.25% trypsin EDTA solution (Thermo Fisher, 25200056). After an incubation of 5 min at 37 °C, 9 ml of medium was added and the cells were resuspended and transferred to a Falcon centrifugal tube. Cells were centrifuged (4 °C, 0.3*g*, 5 min) and the supernatant was removed. The cell pellet was either stored at −20 °C or directly used for DNA extraction. gDNA was extracted from cell pellets using the Monarch gDNA extraction kit (NEB, T3010L), following the manufacturer’s instructions. In the case of oxidative-modification analysis, DPBS used in the cell pellet resuspension step was supplemented with 1 mM deferoxamine (Sigma-Aldrich, D9533) and 50 µM *n*-*tert*-butyl-α-phenylnitrone (Sigma-Aldrich, B7263) to avoid artifactual oxidation. Extracted gDNA was quantified using the Quantus fluorometer with QuantiFluor ONE dsDNA dye (Promega, E4870). Sonication of gDNA was performed using a Q800R2 sonicator (QSonica) with the following settings: 4 °C, 20% amplitude and pulse 15 s on and 5 s off for a total 5 min. The resulting fragments were analyzed on an Agilent 2200 TapeStation using a high-sensitivity D100 ScreenTape.

### Fluorescence labeling of DNA modifications in oligonucleotides

For 8-oxoG site labeling, preannealed oligonucleotide DNA (IL1-T30, IL-1_oxoG; Supplementary Table 1) was used in concentrations of 2, 1, 0.75 and 0.5 µM. For the negative control, 2 µM preannealed oligonucleotide DNA (IL1-T30, IL-1; Supplementary Table 1) was used. DNA was mixed with 1 µl of FPG (NEB, M0240L) and 1 µl of T4 PNK (NEB, M0236L) in 1× NEBuffer 2 (NEB, B7002S) in a final volume of 10 µl. The reaction mixture was incubated for 1 h at 37 °C directly followed by the addition of 1.5 µl of prop-dGTP (Jena Biosciences (custom synthesis), 2.5 mM), 0.5 µL NEBuffer 2 (NEB, B7002S), 2.8 µl of H_2_O and 0.2 µl of Therminator IX (NEB (custom synthesis), 10 U per µl) and incubated for 10 min at 60 °C. DNA was purified using the Monarch PCR & DNA cleanup kit (NEB, T1130L) according to the manufacturer’s instructions using the oligonucleotide protocol and an elution volume of 6 µl. The CuAAC reaction was performed adding 4 µl of AF594 picolyl azide (Jena Biosciences, CLK-1296-AZ-1, 10 mM stock in DMSO), 4 µl of DMSO (Sigma-Aldrich, D8418), 2 µl of premixed CuSO_4_:THPTA (THPTA from Lumiprobe, F4050; CuSO_4_ from Sigma-Aldrich, C1297; 20 mM Cu^2+^ and 200 mM THPTA) and 2 µl of sodium phosphate buffer (Sigma-Aldrich, 71643 and 71505; 1 M, pH 7.0). The reaction was started by adding 2 µl of freshly prepared sodium ascorbate (Sigma-Aldrich, 1613509; 400 mM in H_2_O, freshly prepared) and incubated for 60 min at 37 °C. The reaction was purified using the Monarch PCR & DNA cleanup kit with 20 µL for elution. Then, 8 µl of each sample was used for analysis by 20% (w/v) denaturing PAGE, performed at 230 V for 1 h. After imaging using a ChemiDoc XRS+ System (Bio-Rad), gels were stained with 1× GelRed (Biotium, 41003) for 10 min and imaged again. For fluorescence analysis, the remaining oligonucleotide DNA was mixed with 600 µl of PBS. The sample was split in 3 × 200 µl and pipetted in a nonclear 96-well black plate. For blank measurement, 3 × 200 µl of PBS was added to the plate. Fluorescence was recorded with an Infinite Pro M200 Plate Reader (Tecan) using the following settings: λ_Ex_ = 580 nm, λ_Em_ = 610 nm, gain = optimal (or kept identical for replicates), 25 flashes, 20 µs of integration time and 25 °C.

### Blocking and repair of DNA modifications

A total of 4 µg of gDNA was used for each sample. To minimize artifactual damage, samples were inverted or flicked instead of pipette mixing. Pronex magnetic bead (Promega, NG2002) purification was performed by adding 80 µl of beads to a sample of 50 µl (adjusted with H_2_O), following the manufacturer’s instructions and eluting with Buffer EB. For ddNTP blocking, gDNA was first incubated with 1 µl of ENDOIV (NEB, M0304L) in 1× ThermoPol buffer (NEB, B9004S) in a final volume of 20 µl for 20 min at 37 °C. Then, 2.5 µl of ddNTP mix (Jena Biosciences, NU-1019S), 0.5 µl of 10× ThermoPol buffer (NEB, B9004S), 1.8 µl of H_2_O and 0.2 µl of Therminator IX (NEB (custom synthesis) 10 U per µl) was added, followed by a 10-min incubation at 60 °C. The repair mix modified from Zatopek et al. comprised gDNA with 1 mM NAD^+^ (NEB, B9007S), 1 µl of dNTP mix (NEB, N0447S), 1 µl of ENDOIV (NEB, M0304L), 5 µl of TAQ ligase (NEB, M0208S), 0.5 µl of BST DNA polymerase FL (NEB, M0328S) and 2 µl of 10× ThermoPol buffer (NEB, B9004S) in a total volume of 20 µl and was incubated for 30 min or overnight at 37 °C. The repair mix from Shu et al. contained 2 μl of ENDOIV (NEB, M0304L), 1 μl of BST DNA polymerase FL (NEB, M0328S), 2 μl of TAQ ligase (NEB, M0208S), 1 μl of NAD^+^ (NEB, B9007S), 1 μl of dNTP mix (NEB, N0447S, 10 mM) and 2 µl of 10× NEBuffer 3 (NEB, B7003S) in a total volume of 20 µl and was incubated for 40 min at 37 °C and 60 min at 45 °C. gDNA was purified using Pronex magnetic beads as described previously and eluted in 20 µl.

### HPLC–MS/MS analysis of 8-oxodG in gDNA

A total of 40 µg of gDNA was used for each sample. Digestion mix was prepared for each sample in a total volume of 50 µl. First, 2.5 mM deferoxamine (Sigma-Aldrich, D9533), 2.5 mM *n*-*tert*-butyl-α-phenylnitrone (Sigma-Aldrich, B7263), 20 mM Tris-HCl (Sigma-Aldrich, T5941; pH 7.9), 20 mM MgCl_2_ (Sigma, M8266), 100 mM NaCl (Sigma-Aldrich, S9888) and LC–MS-grade H_2_O were mixed. To this mixture, 50 U of Benzonase nuclease (Sigma, E1014-5KU), 0.06 U of PDE I (US Biologicals, P4072; 0.1 U per µl), 40 U of Antarctic phosphatase (NEB, M0289S) and 100 pg of [^15^N_5_]8-oxodG (Cambridge Isotope Laboratories, NLM-6715-1.2) were added and gently mixed. Then, 50 µl of digestion mix was added to 150 µl of gDNA and incubated for 4 h at 37 °C while shaking at 300 rpm. Digested samples were loaded on a prewashed filter unit (10-kDa molecular weight cutoff; VWR) and centrifuged (16,500*g*, 10 min). For total dG quantification, 2 µl of flowthrough was taken. The remaining flowthrough was used for 8-oxoG enrichment. Solid-phase extraction (SPE) was performed for 8-oxodG enrichment on a vacuum manifold (Visiprep 24 DL from Supelco). SPE columns (Strata X 33M Polymeric RP, 30 mg/1 ml tubes (8B-S100-TAK) from Phenomenex) were activated twice with 1 ml of methanol and twice with 1 ml of LC–MS-grade H_2_O. The remaining flowthrough from the previous step was loaded on the activated columns. Columns were washed twice with 1 ml of LC–MS-grade H_2_O. Enriched samples were eluted in 20% methanol and concentrated to dryness using vacuum centrifugation (miVAC concentrator, DUC-23050-D00). Dried samples were redissolved in 25 μl of H_2_O and sonicated for 10 min (Telsonics Ultrasonics, TPC-120).

The LC–nanoelectrospray ionization (NSI) MS/MS was performed on a TSQ Quantiva triple-quadrupole MS instrument (Thermo Fisher Scientific) coupled to an Acquity UPLC M-Class (Waters) system using NSI. The analysis was conducted using a capillary column (inner diameter: 150 µm, packing length: 5.5 cm, orifice: 15 µm) created by hand-filling a commercially available fused-silica emitter (MSWIL, Aarle-Rixtel) with HSS T3 separation medium (Waters). The mobile phase consisted of 0.1% (v/v) formic acid in water (A1) and 0.1% (v/v) formic acid in acetonitrile (B1). A 1-µl injection loop was used and the sample (1 µl) was loaded onto the capillary column with 2 µl min^−1^ flow in the initial conditions (95% A1, 5% B1) for 2 min and eluted with a linear gradient at a flow rate of 2 µl min^−1^ over 3 min to 60% A1, following by ramping to 99% B1 within 2 min and holding at this composition for an additional 1 min. The column was then reequilibrated at the initial conditions for 3 min before the next injection. The nanoelectrospray source was operated in positive ion mode with the voltage set at 2.7 kV. The ion transfer tube temperature was 250 °C and the radiofrequency lens was used as calibrated. The collision gas was argon at 1.5 mTorr with collision energy of 22 eV and the quadrupoles were operated at a resolution of 0.7 Da for both Q1 and Q3. The mass transitions for monitoring the analytes were *m*/*z* 268.1 → 152.1 for dG, *m*/*z* 284.1 → 168.1 for 8-oxodG and *m*/*z* 289.1 → 173.1 for [^15^N_5_]8-oxodG.

The quantitation of the analytes was performed using the MS vendor software package Quan Browser in the software suite Xcalibur on the basis of the peak areas and the constructed calibration curves. Calibration curves were constructed for each analyte during each analysis using a series of standard solutions of analytes. Calibration curves for dG and 8-oxodG were prepared with concentrations of 200, 175, 150, 75, 50 and 25 nM and 75, 50, 40, 30, 20 and 15 nM, respectively.

### Fluorescence labeling of DNA modifications in gDNA (click-fluoro-quant)

If cells were treated with irofulven, adducts were converted to AP sites by diluting 4 μg of gDNA with H_2_O to a final volume of 15 μl and incubating for 18 h at 37 °C. The choice of enzymes in the following step depends on the lesion of interest as described in the main text. Enzymes can be replaced by H_2_O if the analysis of a specific lesion is not desired. To convert DNA modifications to 3′-OH sites, a combination of 1 µl of FPG (NEB, M0240S), 1 µl of ENDOIV (NEB, M0304L) and 1 µl of T4 PNK (NEB, M0236L) was mixed with 4 µg of gDNA (or a sample from previous blocking and repair step) in 1× NEBuffer 2 (NEB, B7002S) in a final volume of 20 µl. The reaction mixture was incubated for 1 h at 37 °C, immediately followed by adding 3 µl of prop-dGTP (Jena Biosciences (custom synthesis), 2.5 mM), 3 µl of 10× ThermoPol buffer (NEB, B9004S), 3 µl of H_2_O and Therminator IX (NEB (custom synthesis), 10 U per µl) to the sample. In case of quantifying AP sites, 3 µl of H_2_O was replaced by 3 µl of prop-dATP (Jena Biosciences, custom synthesis, 2.5 mM). After 10 min of incubation at 60 °C, gDNA samples were purified using Pronex magnetic beads as described previously with an elution volume of 30 µl. For CuAAC, the sample volume was condensed to 6 µl using a vacuum concentrator. The reaction was performed with 4 µl of AF594 picolyl azide (Jena Biosciences, CLK-1296-AZ-1, 10 mM stock in DMSO), 4 µl of DMSO (Sigma-Aldrich, D8418), 2 µl of premixed CuSO_4_:THPTA (THPTA from Lumiprobe, F4050; CuSO_4_ from Sigma-Aldrich, C1297; 20 mM Cu^2+^ and 200 mM THPTA)^91^ and 2 µl of sodium phosphate buffer (Sigma-Aldrich, 71643 and 71505; 1 M, pH 7.0). The reaction was started by adding 2 µl of sodium ascorbate (Sigma-Aldrich, 1613509; 400 mM in H_2_O, freshly prepared) and incubated for 60 min at 37 °C. The reaction was purified using the Monarch gDNA purification kit (NEB, T3010L), and eluted in 100 µl of buffer EB. gDNA concentration was measured using the Quantus fluorometer with QuantiFluor ONE dsDNA dye (Promega, E4870). For fluorescence analysis, identical amounts of each gDNA sample were mixed with PBS for a final volume of 605 µl. The samples were split into 3 × 200 µl and pipetted in a nonclear 96-well black plate. For blank measurement, 3 × 200 µl of PBS was added to the plate. Fluorescence was recorded with an Infinite Pro M200 plate reader (Tecan) using the following settings: λ_Ex_ = 580 nm, λ_Em_ = 610 nm, gain = optimal, 25 flashes, 20 µs of integration time and 25 °C. The same gain value was used for replicates of the same experiment and usually set to 255. Data were analyzed by subtracting the median blank fluorescence from all sample fluorescence values. They were further normalized to the DNA amount by dividing each value by the amount of gDNA in the sample in ng. The resulting values were rescaled by min–max normalization using the formula ${d}_{\mathrm{norm}}=\frac{d-{d}_{\min }}{{d}_{\max }-{d}_{\min }}$, where $d$ is a data point, ${d}_{\min }$ is the lowest value in a dataset and ${d}_{\max }$ is the highest value in a dataset.

### Click-fluoro-quant plotting and statistics

For all plots concerning click-fluoro-quant and related statistical analyses, we used Jupyter notebooks with the modules numpy version 1.22.4, scipy version 1.8.1, pandas version 1.4.2, matplotlib version 3.5.2 and seaborn version 0.11.2 in Python version 3.10.4 (GCC 8.2.0). The code is available online (https://gitlab.ethz.ch/eth_toxlab/click-code-seq/, folder: click-fluoro-quant).

### Plasmid construction with site-specific 8-oxoG and uracil

Plasmids containing site-specific 8-oxoG and uracil were constructed by inserting double-stranded oligonucleotides into a 4.7-kbp plasmid backbone using a restriction–ligation strategy. The plasmid pEGFP-W (ref. ^18^), derived from pEGFP-N1 (Addgene, 6085-1), was purified from BL21(DE3) *Escherichia* *coli* (NEB, C2527H) using a Qiagen plasmid maxi prep kit (Qiagen, 12162) and digested with BamHI-HF (NEB, R3136S) and HindIII-HF (NEB, R3104S) restriction enzymes, generating sticky ends for subsequent ligation. Specifically, 20 U of each enzyme was added to 5 µg of the plasmid along with 5 µl of 10× rCutsmart buffer (NEB, B6004S) in a final volume of 50 µl, followed by incubation for 1 h at 37 °C. The digested plasmid backbone was purified using the Monarch spin PCR & DNA cleanup kit (NEB, T1130S) to remove the excised 33-bp fragment. For the 8-oxoG modified plasmid, a pair of 5′-phosphorylated oligonucleotides (For_oxoG and Rev_oxoG; Supplementary Table 1) was designed with complementary 4-nt overhangs corresponding to BamHI and HindIII sticky ends (Extended Data Fig. 2g). The reverse strand of this pair contained an 8-oxoG modification at a specific site. For the uracil-modified plasmid, an analogous pair of oligonucleotides (For_U and Rev_U; Supplementary Table 1) were synthesized, where the reverse strand contained a uracil at the same site. All oligonucleotides were synthesized by Eurogentec. Annealing of the complementary oligonucleotides was conducted by mixing them at equimolar concentrations and gradually cooling the mixture from 95 °C to room temperature. The annealed products were ligated into 500 ng of the digested plasmid at an vector-to-insert molar ratio of 1:20 using 2.5 µl of T4 DNA ligase (NEB, M0202S) and 5 µl of 10× T4 DNA ligase reaction buffer (NEB, B0202S) in a final volume of 100 µl. Ligation reactions were incubated at 16 °C overnight and performed in ten parallel replicates for each construct to maximize yield. Successfully ligated plasmids were identified by agarose gel electrophoresis, where they migrated at the same position as the undigested plasmid control, purified using the Monarch spin gel extraction kit (NEB, T1120S) and quantified using a Quantus Fluorometer. We verified the presence of the new sequence using Sanger sequencing with the primer For_pEGFP (Supplementary Table 1). The ligation procedure yielded 72 ng and 60 ng of the 8-oxoG and uracil-modified plasmids, respectively, corresponding to ~25 fmol and ~21 fmol of the site-specific DNA modifications. Each modified plasmid was separately spiked into 2 µg of the original pEGFP-W plasmid and subjected to click-code-seq library preparation (described below). In the case of the uracil-modified plasmid, UDG (NEB, M0280S) was used instead of FPG to excise the uracil base. Two control samples containing 2 µg of unmodified pEGFP-W plasmid were processed under identical conditions with either FPG or UDG.

### Click-code-seq library preparation

To minimize artefactual damage, samples were inverted or flicked, instead of pipette mixing, until the CuAAC step. In case of DNA oxidation mapping, gDNA was eluted in H_2_O supplemented with 500 µM 8-hydroxyquinoline (Sigma-Aldrich, 252565) after extraction and purification. Undesired background modifications were blocked by adding 1 µl of ENDOIV (NEB, M0304L) and 500 µM 8-HQ to 5 µg of gDNA, freshly extracted from HAP1 pellets, in 1× ThermoPol buffer (NEB, B9004S) in a final volume of 20 µl, and incubating the mixture for 20 min at 37 °C. Then, 2.5 µl of ddNTP mix (Jena Biosciences, NU-1019S2.5 mM), 0.5 µl of 10× ThermoPol Buffer (NEB, B9004S), 1.8 µl of 8-HQ (500 µM) and 0.2 µl of Therminator IX (NEB (Custom synthesis), 10 U per µl) were added, followed by a 10-min incubation at 60 °C. Next, 25 µl of 8-HQ (500 µM) and 80 µl of Pronex beads (Promega, NG2002) were added for purification with an elution volume of 25 µl. To label oxidative modifications, 1 µl of FPG (NEB, M0240L) 1 µl of ENDOIV (NEB, M0304L) and 3 µl of 10× NEBuffer 2 (NEB, B7002S) were added for a final volume of 30 µL; the reaction mixture was incubated for 1 h at 37 °C, immediately followed by adding 3.5 µl of prop-dGTP (Jena Biosciences (custom synthesis), 2.5 mM), 0.5 µl of 10× NEBuffer 2 (NEB, B7002S), 0.8 µl of 8-HQ (500 µM) and 0.2 µl of Therminator IX (NEB (custom synthesis), 10 U per µl) for 10-min incubation at 60 °C. As for the plasmid DNA, each modified plasmid was separately spiked into 2 µg of the original pEGFP-W plasmid and subjected to these same initial steps. In the case of the uracil-modified plasmid, UDG (NEB, M0280S) was used instead of FPG to excise the uracil base and prop-dTTP (Jena Biosciences (custom synthesis), 2.5 mM) was used instead of prop-dGTP. Two control samples containing 2 µg of unmodified pEGFP-W plasmid were processed under identical conditions with either FPG and prop-dGTP or UDG and prop-dTTP.

In case of mapping AP sites, 5 µg of freshly extracted U2OS gDNA was diluted with water to a final volume of 20 µl and incubated at 37 °C for 18 h; then, to dephosphorylate background strand breaks, 1 µl of T4 PNK (NEB, M0236L) was added to the gDNA in 1× NEBuffer 2 (NEB, B7002S) in a final volume of 25 µl and incubated for 30 min at 37 °C. Next, 3 µl of ddNTP mix (Jena Biosciences, NU-1019S, 2.5 mM), 0.5 µl of 10× NEBuffer 2 (NEB, B7002S), 1.3 µl of water and 0.2 µl of Therminator IX (NEB (custom synthesis), 10 U per µl) were added, followed by a 10-min incubation at 60 °C. Then, 20 µl of water and 80 µl of Pronex beads (Promega, NG2002) were added for purification with an elution volume of 25 µL. To label AP sites, 1 µl of ENDOIV (NEB, M0304L) and 3 µl of 10× ThermoPol buffer (NEB, B9004S) were added for final volume of 30 µl; the reaction mixture was incubated for 30 min at 37 °C, immediately followed by adding 4 µl of prop-dGTP (Jena Biosciences (custom synthesis), 2.5 mM in H_2_O), 5 µl of prop-dATP (custom synthesis), 2 mM in H_2_O), 1 µl of 10× ThermoPol buffer (NEB, B9004S), 0.8 µl of H_2_O and 0.2 µl of Therminator IX (NEB, (custom synthesis), 10 U per µl) for a 10-min incubation at 60 °C.

Steps for mapping either AP sites or oxidative damage, both for gDNA and plasmid DNA, are identical from this point onward. DNA was purified using Pronex magnetic beads (Promega, NG2002) with a sample-to-bead ratio of 50:80 and using an elution volume of 50 µl with buffer EB. Sonication of DNA was performed using a Q800R2 sonicator (QSonica) with the following settings: 4 °C, 20% amplitude and pulse 15 s on, 5 s off for a total of 5 min for gDNA and 4 min for plasmid DNA. The resulting DNA fragments were analyzed on a TapeStation 2200 using a high-sensitivity D100 ScreenTape (Agilent Technologies). DNA was purified using Pronex beads with a sample-to-beads ratio of 1:2 and 50-µl elution volume. Samples were quantified using a Quantus fluorometer. For adaptor ligation, the NEBNext Ultra II DNA library prep kit for Illumina (NEB, E7645S) was used with either 1 µg of gDNA or the remaining plasmid DNA and the preannealed NEB adaptor (NEB_P7, NEB_P5, 15 µM; Supplementary Table 1). The manufacturer’s instructions were followed without performing the USER enzyme step. The sample was purified using Pronex beads in a sample-to-beads ratio of 1:2 and elution in 50 µl of buffer EB. Successful adaptor ligation was checked on an Agilent TapeStation with a high-sensitivity D100 ScreenTape. The sample volume was then condensed to 6 µl using a vacuum concentrator. To denature DNA, the sample was heated to 95 °C for 2 min and rapidly cooled on ice. The CuAAC reaction was performed using 2 µl of MoDIS (100 µM equimolar mixture of MoDIS_1 and MoDIS_2; Supplementary Table 1), 4 µl of DMSO (Sigma-Aldrich, D8418), 2 µl of premixed CuSO_4_:THPTA (THPTA from Lumiprobe, F4050; CuSO_4_ from Sigma-Aldrich, C1297; 20 mM Cu^2+^ and 200 mM THPTA) and 2 µl of sodium phosphate buffer (Sigma-Aldrich 71643 and 71505; 1 M, pH 7.0). The reaction was started by adding 2 µl of sodium ascorbate (Sigma-Aldrich, 1613509; 400 mM in H_2_O, freshly prepared) and incubated for 60 min at 37 °C. Purification was performed using Pronex beads in a sample-to-beads ratio of 1:2 and 30-µl elution volume. Biotin enrichment of sample DNA was performed using Dynabeads MyOne Streptavidin C1 (Thermo Fisher, 65001). First, B&W buffer was prepared according to the manufacturer’s protocol including 0.05% Tween-20 (Sigma-Aldrich, 11332465001). Then, 15 µl of Dynabeads were transferred to a fresh tube, washed three times with 200 µl of 1× B&W buffer and resuspended in 30 µl of 2× B&W buffer. The DNA sample was denatured by heating to 95 °C for 2 min and rapid cooling on ice. Then, 30 µl of sample was mixed with 30 µl of the prepared Dynabeads and rotated for 15 min at room temperature. Afterwards, the Dynabeads were washed three times with 1× B&W buffer by resuspending the beads, followed by 3 min of rotation and 1 min of pelleting on a magnetic rack. A final wash was performed with buffer EB and Dynabeads were resuspended in 20 µl of Buffer EB. To amplify the biotin-enriched material, a PCR was performed by adding 5 µl of 10× ThermoPol buffer (NEB, B9004S), 5 µl of dNTP mix (NEB, N0447S, 2 mM), 1 µl of MgSO_4_ (NEB, B1003S), 1 µl of ex_UMI (10 µM; Supplementary Table 1), 1 µl of Pr_P7 (10 µM; Supplementary Table 1) and 0.5 µl of Vent (exo-) DNA Polymerase (NEB, M0257L) The PCR program comprised an initial denaturation step (95 °C, 120 s), followed by five cycles of denaturation (95 °C, 20 s), annealing (59 °C, 20 s) and elongation (72 °C, 60 s) and a final elongation step (72 °C, 300 s). The sample was purified with Pronex® beads in a sample-to-beads ratio 1:2 and an elution volume of 30 µl. Final library amplification and indexing were performed by adding 25 µl of NEBNext Ultra II Q5 master mix (NEB, M0544L), 2.5 µl of i5_Index (10 µM; Supplementary Table 1) and 2.5 µl of i7_Index (10 µM; Supplementary Table 1) to 20 µl of sample. The PCR program comprised an initial denaturation step (98 °C, 30 s), ten cycles of denaturation (98 °C, 10 s), annealing (68 °C, 30 s) and elongation (72 °C, 20 s) and a final elongation step (72 °C, 120 s). The same PCR setup can be used for a precedent qPCR by adding a qPCR dye as Evagreen (Biotium, 31000) to test for successful sample amplification. The PCR sample was purified using the dual-size selection protocol with Pronex beads at an initial sample-to-beads ratio of 1:1, followed by a sample-to-beads ratio of 0.5:1 to exclude fragments longer than 1,000 bp and shorter than 250 bp. The final sample concentration was adjusted to a concentration of 4 ng µl^−1^ and sequenced on an Illumina NovaSeq 6000.

### Click-code-seq data analysis

#### Sequencing read processing

After demultiplexing of sequencing data, each sample was represented by a fastq.gz file containing 101-nt-long genomic reads. The quality of the raw sequencing data was checked using FastQC version 0.11.9 or 0.12.1 (GSK-3484862 experiment, GE; plasmids, P). Low-quality reads and adaptor-containing reads were removed using trimmomatic version 0.38 or 0.39 (GE, P). We retained only those reads that contained a VC. The first 17 nt corresponding to the VC and RIC were clipped from the read sequences and appended to the read names using the tool extract of umi_tools toolkit version 1.1.2 or 1.1.4 (GE, P). In the case of samples from cells, reads were mapped to human reference genome GRCh38 using bowtie2 version 2.3.5.1 or 2.5.1 (GE) using a prebuilt bowtie2 index (https://genome-idx.s3.amazonaws.com/bt/GRCh38_noalt_as.zip) and applying otherwise standard settings. In the case of samples originating from plasmids, we built bowtie2 indices for three plasmids: (1) input plasmid (vector); (2) plasmid with triple 8-oxoG insert, 8-oxoG represented by G; and (3) plasmid with triple U insert, U represented by T (triple insert explained in Extended Data Fig. 2b). The plasmid sequences were flanked by 100 nt to mimic a circular structure in read alignment; these indices were used to map reads with bowtie2 2.5.1. Read duplicates were removed by the tool dedup of umi_tools toolkit version 1.1.2 or 1.1.4 (GE, P), grouping reads with the same code sequence stored in the read name (method = unique). SAMtools version 1.12 or 1.17 (GE, P) was used to sort, index and generate statistics of BAM files. BEDTools2 version 2.29.2 or 2.31.0 (GE, P) was used to retrieve the coordinates of mapped and deduplicated reads and extract the sequence context of DNA modifications from the reference genome. Each read represented one unit of DNA-modification signal, which we positioned at the nucleotide of the 5′ end of the read. Because a read was the reverse complement of the DNA fragment captured in the method using MoDIS, the strand of the nucleotide bearing the signal was changed to the opposite one. In GE, we retained reads with mapping quality higher than 40 for downstream analysis. Supplementary Fig. 4 describes the evolution of read counts throughout the preprocessing steps. We implemented the described DNA-modification-signal positioning using custom scripts in Python version 3.7.4 (GCC 4.8.5) with the modules numpy version 1.21.5, pandas version 0.25.1 and biopython version 1.79 or, for GE and P, in Python version 3.11.6 (GCC 12.2.0) with modules numpy version 1.25.2, pandas version 2.2.2 and biopython version 1.83.

#### Software for mapped DNA-modification data analysis

The downstream analysis of DNA-modification data and their visualization were performed in Jupyter notebooks with the modules numpy version 1.26.4, scipy version 1.13.1, pandas version 2.2.2, biopython version 1.83, matplotlib version 3.9.0, seaborn version 0.13.2, pyBigWig version 0.3.23 and pyfaidx version 0.8.1.1 in Python version 3.11.6 (GCC 13.2.0), in Jupyter notebooks with the modules numpy version 1.22.4, scipy version 1.8.1, pandas version 1.4.2, biopython version 1.79, matplotlib version 3.5.2, seaborn version 0.11.2, logomaker version 0.8 and pyfaidx version 0.6.4 in Python version 3.10.4 (GCC 8.2.0), in Jupyter notebooks with the modules numpy version 1.19.2 and pandas version 1.1.3 in Python version 3.8.5 (GCC 6.3.0), in custom scripts in Python version 3.7.4 (GCC 4.8.5) with the modules numpy version 1.21.5, pandas version 0.25.1 and biopython version 1.79 or in custom scripts in Python version 3.11.6 (GCC 12.2.0) with the modules numpy version 1.25.2, pandas version 2.2.2 and biopython version 1.83. The custom code for analyzing click-code-seq data and plotting figures is available online (https://gitlab.ethz.ch/eth_toxlab/click-code-seq).

#### External datasets

The human reference genome GRCh38 (https://genome-idx.s3.amazonaws.com/bt/GRCh38_noalt_as.zip) (bowtie2 prebuilt index) was used. For the analysis related to gene expression and chromatin accessibility, we used the data from GRCh38 chr1–22 and chrX. SBS signatures were from COSMIC version 3.4 (https://cancer.sanger.ac.uk/signatures/documents/2124/COSMIC_v3.4_SBS_GRCh38.txt). The illudin S mutational signature was from a previous study^43^ (https://ars.els-cdn.com/content/image/1-s2.0-S1568786422001665-mmc1.xlsx; Supplementary material, Tab_S4, ILS Clones, % of SBS (count no background), ILS Sig). Centromeres and gaps were from the UCSC Table Browser for GRCh38. Chromatin accessibility and histone marks were from ChIP-Atlas (https://chip-atlas.org/peak_browser), with a significance threshold 50, downloaded on February 13, 2024, with the following accession numbers: for U2OS, H3K4me1, GSM1901957 and GSM4861712; H3K9me3, GSM4079837, GSM3147771 and GSM788078; H3K4me3, GSM2341637 and GSM3147770; H3K36me3, GSM788076; H3K27ac, GSM2341636, GSM6836721, GSM4133285, GSM4133286, GSM5018581, GSM5018582 and GSM6836725; DNase hypersensitive sites, GSM4221655; for HAP1, H3K4me1, GSM3579008; H3K9me3, GSM3579026; H3K4me3, GSM5570286, GSM2978165, GSM3901520, GSM2978166 and GSM3901519; H3K36me3, GSM5570287; H3K27me3, GSM5570291, GSM2978164, GSM3901518, GSM3901517, GSM2978163, GSM5770668 and GSM5770667; H3K27ac, GSM3901521 and GSM3901522; DNase hypersensitive sites, GSM5214993, GSM5214992, GSM2400413 and GSM2400414. U2OS MNase-seq data were from GSM1838916 (GSM1838916_NT1.wig.gz) and GSM1838917 (GSM1838917_NT2.wig.gz). Repli-seq data for HAP1 were from GSE160563 (GSE160563_HAP_WT_log2EL_RT.bigwig) and for U2OS were from GSE196749 (GSE196749_Optimized_Repli-seq_Human.txt.gz). Transcript coordinates were from GENCODE/V41/knownGene, obtained from the UCSC Table Browser. Canonical transcripts of genes were from GENCODE/V41/knownCanonical, obtained from the UCSC Table Browser. Protein-coding genes were from GENCODE/V41/knownToNextProt, obtained from the UCSC Table Browser. Genes were represented by canonical transcripts between the TSS and TES. Gene expression data were from CCLE_expression_full from DepMap Public 22Q2 (https://depmap.org/portal/download/all/), with the cell line accession numbers ACH-002475 (HAP1) and ACH-000364 (U2OS). Mitochondrial gene and D-loop-region annotation was from NC_012920.1 (https://www.ncbi.nlm.nih.gov/nuccore/251831106). EPIC version 2.0 manifest file EPIC-8v2-0_A2.csv was obtained online (https://emea.support.illumina.com/downloads/infinium-methylationepic-v2-0-product-files.html) on January 17, 2025.

#### Correlation between chromatin state and DNA modifications

We used ChIP-Atlas data for relevant cell lines under control conditions (without an exposure or genetic intervention). Average ChIP-seq peak sizes were 118–1,023 bp in HAP1 (Fig. 3a) ad 79–636 bp in U2OS (Fig. 3d,g). Bin numbers were as follows: 28,601 in 100-kbp binning; 285,360 for G modification and 285,354 for A modification in 10-kbp binning; 2,852,127 for G modification and 2,852,122 for A modification in 1-kbp binning. For every bin with its lower and upper boundaries $$b=\left[{b}_{\mathrm{lo}},{b}_{\mathrm{up}}\right]$$ and ChIP-seq mapping study $$z$$ with chromatin-mark peaks defined by the lower and upper boundaries $$p(z)=\left[{p}_{\mathrm{lo}}(z),{p}_{\mathrm{up}}(z)\right]$$, we calculated the peak coverage of the bin $$e(b,z)=$$$$\frac{1}{{b}_{\mathrm{up}}-{b}_{\mathrm{lo}}+1}{\sum }_{p(z):p(z)\cap b\ne \varnothing }[\min \left({p}_{\mathrm{up}}(z),{b}_{\mathrm{up}}\right)-\max \left({p}_{\mathrm{lo}}(z),{b}_{\mathrm{lo}}\right)+1]$$. We excluded bins that did not contain the target nucleotide (G or A) according to the reference genome or overlap with centromeric regions or heterochromatin and short-arm gaps of the reference genome. For each type of DNA modification $$T$$, namely, guanine oxidation, irofulven-induced AP sites at adenines or background AP sites at guanines, we averaged the binned DNA-modification data $${\{\bar{C}\left(T,b\right)\}}_{b}={{{mean}}_{s\in T}\{C\left(s,T,b\right)\}}_{b}$$, where $$s$$ is a replicate experiment and $$C\left(s,b\right)$$ is the DNA-modification level normalized per bin to the reference genome and sequencing depth, as described below. For each ChIP-seq mapping study $$z$$ and DNA-modification type $$T$$, we then calculated the coefficient of Spearman correlation $$\rho (z,T)$$ between the binned chromatin-mark data $${\{e\left(b,z\right)\}}_{b}$$ and the averaged binned DNA-modification data $${\{\bar{C}\left(T,b\right)\}}_{b}$$. $$\rho (z,T)$$ values are presented as markers in Fig. 3a,d,g, with the bar being the median across the mapping studies $$z$$.

#### Integration of DNA-modification and Repli-seq data

We decreased the resolution of U2OS Repli-seq data (GSE196749_Optimized_Repli-seq_Human.txt.gz) from 10 kbp to 50 kbp by averaging to match the 50-kbp resolution of the available HAP1 Repli-seq dataset (GSE160563_HAP_WT_log2EL_RT.bigwig). We used the replicate U2OS_OptRepliseq_R3_hg38 for further analysis as it had the highest pairwise correlation among replicate Repli-seq measurements in U2OS cells. We excluded the HAP1 and U2OS Repli-seq data corresponding to centromeric regions and heterochromatin and short-arm gaps of GRCh38 assembly. To correlate the log_2_ EL_RT (early/late replication timing) signal with the levels of DNA modifications, they were aggregated at 50-kbp resolution and normalized by the respective counts of target nucleotides in the reference genome and by normalization factors, as described below. To identify the leading-strand and lagging-strand templates, we performed the following steps. First, we identified >300-kbp regions where the 50-kbp slopes of log_2_ EL_RT signal were continuously positive or negative. Second, we determined the 25th percentile ${p}_{25}$ of the distribution of the absolute values of the 50-kbp slopes in the monotonic >300-kbp regions and used this as a threshold to identify >250-kbp regions where the 50-kbp slopes are continuously bigger than ${p}_{25}$ or smaller than $-{p}_{25}$, with this filtering aimed to remove breakage points and obtain true timing transition regions (TTRs) hypothesized to reflect unidirectional replication forks^92^. Third, in the resulting >250-kbp regions, we removed the last data point adjacent to replication termination regions, such that the final TTRs spanned the areas of >200 kbp. The leading-strand template was then defined as the + strand in the leftward (positive slope) TTRs and the − strand in the rightward (negative slope) TTRs. The lagging-strand template was defined as the − strand in the leftward (positive slope) TTRs and the + strand in the rightward (negative slope) TTRs. To analyze replicative strand biases, we used normalized DNA-modification levels at 50-kbp resolution within the thus defined leading-strand and lagging-strand templates of the TTRs.

#### Integration of DNA-modification and MNase-seq data

Nucleosome positions were identified using available MNase-seq data from U2OS (two replicates GSM1838916_NT1.wig.gz and GSM1838917_NT2.wig.gz, hg19 genome assembly) using the dpos command of DANPOS^93^ version 3.0.0 (obtained from https://github.com/sklasfeld/DANPOS3) implemented in the environment with zlib version 1.3, R version 4.3.2, samtools version 1.17, htslib version 1.17 and Python version 3.11.6 (GCC 12.2.0) with the following modules: rpy2 version 3.3.3, numpy version 1.25.2, pysam version 0.22.1 and argparse version 1.1. The ranges of nucleosome and nucleosome-free DNA were lifted over from hg19 to Hg38 using the UCSC liftOver tool with minMatch = 1.0. The mapped oxidized guanines in nucleosome and nucleosome-free DNA were identified using the intersect command of BEDTools version 2.31.0. The sequence composition of the ranges needed to count reference genome occurrences of trinucleotides and the trinucleotide contexts of mapped oxidized guanines were obtained using the getfasta command of BEDTools version 2.31.0.

#### DNA-modification level normalization

DNA-modification levels in genomic features of interest were corrected by respective counts of target nucleotides (G or A) in the reference genome. Additionally, the genome-scale data required normalization to correct for sequencing depth that varied among samples (Supplementary Fig. 4). We, therefore, computed sample-specific normalization factors that reflect the level of DNA modification $T$ (oxidized guanines, deoxyadenosine-derived AP sites or deoxyguanosine-derived AP sites) in unexpressed genes: $M\left(s,T\right)={\mathrm{median}}_{\mathrm{across}{\ f}}{\,\{\frac{N(s,T,\,f\,)}{N\mathrm{ref}\left(T,\,f\,\right)\,}\}}_{f}$, where features $f$ here are the transcribed (antisense) strands of unexpressed protein-coding genes, $N(s,T,f)$ is the count of mapped DNA modifications $T$ per a concrete feature $f$ in the sample $s$ and $N\mathrm{ref}\left(T,f\right)$ is the feature’s count (kilobases) of target nucleotides for $T$ in the reference genome. DNA-modification levels $C\left(s,T,f\right)$ were related to these sample-specific normalization factors in the following way: $C\left(s,T,f\,\right)=\frac{N(s,T,f\,)}{N\mathrm{ref}\left(T,f\right)\,\bullet M(s,T)\,}$ (arb. units), where a feature $f$ is a dinucleotide (Fig. 2d), a chromosome bin with both strands considered together (Fig. 3a,b,d,e,g,h and Supplementary Figs. 2, 5c, 7c,f and 8), a gene’s transcribed strand or a gene’s nontranscribed strand (Fig. 4a,b,e,f,i,j and Extended Data Fig. 5a,b,e,f) or strands of the TSS-adjacent region (Extended Data Fig. 8). DNA-modification replicative strand bias was calculated as $C\left(s,T,{f}_{{lag}}\right)-C\left(s,T,{f}_{{lead}}\right)$, where ${f}_{\mathrm{lag}}$ and ${f}_{\mathrm{lead}}$ are the lagging-strand template and the leading-strand template, respectively, of a 50-kbp bin (Fig. 3c,f,i). DNA-modification transcriptional strand bias was calculated as $C\left(s,T,{f}_{\mathrm{NTS}}\right)-C\left(s,T,{f}_{\mathrm{TS}}\right)$, where ${f}_{\mathrm{NTS}}$ and ${f}_{\mathrm{TS}}$ are the nontranscribed and transcribed strands, respectively, of the same feature (Fig. 4d,h,l and Extended Data Fig. 5d,h). For metaprofiles (Fig. 4c,g,k and Extended Data Figs. 4b–d and 5c,g), we presented the ${\mathrm{mean}}_{\mathrm{across}{\ g}\in G}{\{C\left(s,T,g,t,b\right)\}}_{g}$ with $C\left(s,T,g,t,b\right)=\frac{N\left(s,T,g,t,b\right)}{N\mathrm{ref}\left(T,g,t,b\right)\bullet M\left(s,T\,\right)}$, where $N(s,T,g,t,{b})$ is the count of mapped DNA modifications $T$ per bin $b$ in sample $s$, gene $g$ and strand $t$, $N\mathrm{ref}\left(T,g,t,b\right)$ is the bin’s count (kilobases) of target nucleotides for $T$ in reference genome in gene $g$ and strand $t$, $M\left(s,T\right)$ is the normalization factor defined above and $G$ is the set of 30% most expressed or unexpressed protein-coding genes. In trinucleotide-specific analysis (Extended Data Figs. 4a, 6 and 7), DNA-modification levels $C\left(s,T,g,t\right)$ were related to the maximal median value within a sample (the maximum of the median values for each sample shown in the plots is set to 1): $C\left(s,T,g,t\right)/{\max }_{\mathrm{across}\left(G,t\right)}{\{{\mathrm{median}}_{\mathrm{across}g\in G}{\left\{C\left(s,T,g,t\right)\right\}}_{g}\}}_{G,t}$ where $g$ is a gene and $t$ is a strand, such that occurrences of only a certain trinucleotide within this gene and strand are considered, $G$ is a gene expression tier and $(G,t)$ is a combination of gene expression tier and strand. In mtDNA analysis (Fig. 5, Extended Data Fig. 9d), DNA-modification level was calculated as follows: $C\left(s,T,t,b\right)=\frac{N\left(s,T,t,b\right)}{N\mathrm{ref}\left(T,t,b\right)\bullet W\left(s,T\right)}$, where $N(s,T,t,{b})$ is the count of mapped DNA modifications $T$ per bin $b$ in sample $s$ and strand $t$, ${Nref}\left(T,t,b\right)$ is the bin $b$’s count (bases) of target nucleotides for $T$ in strand $t$ according to the reference genome and $W\left(s,T\right)$ is the total count of mapped DNA modifications $T$ in mtDNA in sample $s$. In the analysis of plasmids with site-specific DNA modifications and the related input vector (Extended Data Fig. 2c–f), DNA-modification level was calculated as follows: $C\left(s,T,{t},{p}\right)=\frac{N\left(s,T,t,{p}\right)}{\bar{M}\left(s,T\right)\,}$, where $N(s,T,t,{p})$ is the count of DNA modifications $T$ at position (coordinate) $p$ (that is, the number of mapped reads) in sample $s$ and strand $t$, $\bar{M}\left(s,T\right)$ is the median count of mapped DNA modifications $T$ across plasmid positions of both strands in sample $s$. In the analysis of guanine oxidation levels across CpG sites with different methylation (β) values (Extended Data Fig. 3e–h), guanine oxidation level $C\left(s,f\right)$ was calculated as follows: $C\left(s,f\right)=\frac{N(s,f)}{2\bullet N\mathrm{ref}\left(f\right)\,\bullet \,\widetilde{M}(s)\,}$, where $N(s,f\,)$ is the count of guanine oxidation (mapped reads) on both DNA strands in $f$ (all CpG sites with β values within a certain bin (bin width 0.02)) and sample $s$, $N\mathrm{ref}\left(f\right)$ is the number of CpG sites in $f$ divided by 1,000, 2 is to account for two strands at which guanine oxidation is mapped for every CpG site, $\widetilde{M}(s)$ is the sample-specific normalization factor accounting sequencing depth and calculated as ${\mathrm{median}}_{\mathrm{across}{\ b}}{\{\frac{N(s,b)}{N\mathrm{ref}\left(b\right)\,}\}}_{b}$, where $N(s,{b})$ is the count of guanine oxidation in a genomic 100-kbp bin $b$ with both strands considered together and in sample $s$ and $N\mathrm{ref}\left(b\right)$ is the guanine count (kilobases) in the bin with both strands considered together in the reference genome. In the analysis of guanine oxidation levels at CpG sites across different conditions of the DNMT1 inhibition experiment (Extended Data Fig. 3i), guanine oxidation level $C\left(s,f\right)$ was calculated as follows: $C\left(s,f\right)=\frac{N(s,f)}{N\mathrm{ref}\left(f\right)\,\bullet \,\widetilde{M}(s)\,}$, where $N(s,f)$ is the count of guanine oxidation (mapped reads) in $f$ (all CpG sites in the nuclear genome with both strands considered) and in sample $s$, ${Nref}\left(f\right)$ is the total number of CpG sites (divided by 1,000) in the reference genome with both strands considered and $\widetilde{M}(s)$ is the sample-specific normalization factor defined above. In the analysis of guanine oxidation levels across GXX trinucleotides (Supplementary Fig. 6), guanine oxidation level $C\left(s,f\right)$ was calculated as follows: $C\left(s,f\right)=\frac{N(s,f)}{N\mathrm{ref}\left(f\right)\,\bullet \,\widetilde{M}(s)\,}$, where $N(s,f)$ is the count of guanine oxidation (mapped reads) in a trinucleotide $f$ (either throughout the genome, in nucleosome DNA or nucleosome-free DNA) and in sample $s$, $N\mathrm{ref}\left(f\right)$ is the total number of this trinucleotide (divided by 1,000) in the corresponding region of the reference genome and $\widetilde{M}(s)$ is the sample-specific normalization factor defined above.

#### Counts in gene-related analysis

In HAP1, we considered 16,740 protein-coding genes: unexpressed, 3,428; ≤10% expression tier, 1,404; ≤20%, 1,259; ≤30%, 1,332; ≤40%, 1,330; ≤50%, 1,333; ≤60%, 1,329; ≤70%, 1,331; ≤80%, 1,331; ≤90%, 1,331; ≤100%, 1,332. The numbers of data points beyond the maximal *y* axis value were as follows: Fig. 4a, 2,202 (TS) and 2,228 (NTS); Fig. 4d, 48; Extended Data Fig. 8a, 506 (TS) and 493 (NTS). In U2OS, we considered 16,740 protein-coding genes: unexpressed, 1,989; ≤10% expression tier, 1,544; ≤20%, 1,415; ≤30%, 1,467; ≤40%, 1,478; ≤50%, 1,474; ≤60%, 1,474; ≤70%, 1,474; ≤80%, 1,475; ≤90%, 1,475; ≤100%, 1475. The numbers of data points beyond the maximal *y* axis value were as follows: Fig. 4e, 396 (TS) and 526 (NTS); Extended Data Fig. 5e, 2,624 (TS) and 3,112 (NTS); Fig. 4i, 1,116 (TS) and 1,348 (NTS); Extended Data Fig. 5a, 502 (TS) and 528 (NTS); Fig. 4h, 40; Extended Data Fig. 5h, 2,485; Fig. 4l, 251; Extended Data Fig. 5d, 79; Extended Data Fig. 8c, 995 (TS) and 959 (NTS); Extended Data Fig. 8e, 3,239 (TS) and 3,277 (NTS); Extended Data Fig. 8g, 1,319 (TS) and 1,346 (NTS); Extended Data Fig. 8i, 2,361 (TS) and 2,399 (NTS).

### DNA methylation profiling

DNA methylation profiling was performed using the Infinium MethylationEPIC BeadChip version 2.0 at Life & Brain. Specifically, the Standard Illumina Protocol was used for labeling and DNA was bisulfite-converted with the Zymo EZ-96 DNA methylation-lightning kit (D5033), amplified, fragmented and hybridized to human Infinium MethylationEPIC BeadChip version 2.0 (Illumina) using the standard Illumina protocol. Then, arrays were imaged using iScan System and standard recommended Illumina scanning settings. The β values were derived using the R package minfi version 1.50.0. The downstream analysis of β values and their visualization were performed in Jupyter notebooks using the modules numpy version 1.26.4, pandas version 2.2.2 and matplotlib version 3.9.0 in Python version 3.11.6 (GCC 13.2.0).

### Reporting summary

Further information on research design is available in the Nature Portfolio Reporting Summary linked to this article.

## Online content

Any methods, additional references, Nature Portfolio reporting summaries, source data, extended data, supplementary information, acknowledgements, peer review information; details of author contributions and competing interests; and statements of data and code availability are available at 10.1038/s41589-025-02052-6.

## Supplementary information

Supplementary Information Supplementary Figs. 1–8, Table 1, Notes 1–7 and Discussion.

Reporting Summary

## Data Availability

The raw sequencing and DNA-methylation-array data and corresponding processed data (tsv files with called DNA-modification sites, csv file with β values) generated in this study were deposited to the National Center for Biotechnology Information Gene Expression Omnibus (GEO) under the following accession codes: endogenous guanine oxidation in cells and AP sites in irofulven-exposed or DMSO-exposed cells, GSE272366; guanine oxidation in GSK-3484862-exposed or DMSO-exposed cells oxidized guanines and AP sites in plasmids with site-specific DNA modifications, GSE295218; methylation data from GSK-3484862-exposed or DMSO-exposed cells, GSE295295. Supporting data for figures and raw MS data were deposited to Zenodo (10.5281/zenodo.16936776)^94^.

## Code availability

Code used for all analyses and generation of figures was deposited in Zenodo (10.5281/zenodo.16936776)^94^ and is also available from GitLab (https://gitlab.ethz.ch/eth_toxlab/click-code-seq).
