# Supplementary material for: Click-code-seq reveals strand biases of DNA oxidation and depurination in human genome
Source: Nat Chem Biol. 2025 Oct 31;22(5):716–27. doi: 10.1038/s41589-025-02052-6 (PMC13128490; doi:10.1038/s41589-025-02052-6)
Supplement: Supplementary file 1 — Supplementary Figs. 1–8, Table 1, Notes 1–7 and Discussion. [file 41589_2025_2052_MOESM1_ESM.pdf]

nature chemical biology

Supplementary information https://doi.org/10.1038/s41589-025-02052-6

Click-code-seq reveals strand biases of DNA oxidation and depurination in human genome

In the format provided by the authors and unedited

Table of contents:

– Supplementary Figures 1–8	pages 2–11
– Supplementary Table 1	page 12
– Supplementary Notes 1–7	pages 13–20
– Supplementary Discussion 1	pages 21

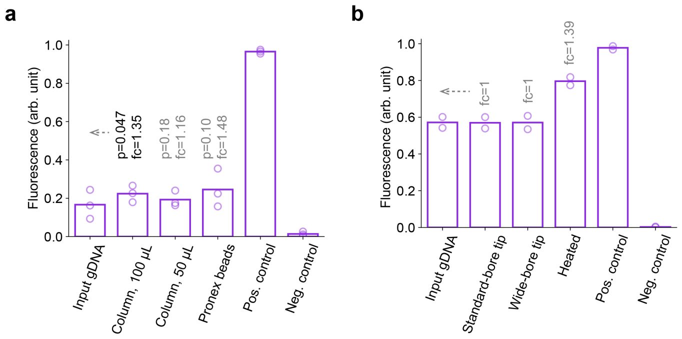

Supplementary Fig. 1. Evaluating DNA-modification levels associated with gDNA-sample-handling factors via click-fluoro-quant. **a** Click-fluoro-quant after gDNA purification with silica columns using two different elution volumes or magnetic beads. **b** Click-fluoro-quant after pipetting gDNA 20 times with a standard-bore or a wide-bore tip, or after incubating gDNA at 85 °C for 20 min (Heated). Bar: mean of n=3 (**a**) or n=2 (**b**) biological replicates (markers); p: p-value of the one-tailed (H_A: greater) paired t-test between the sample under the value and the sample indicated by the arrow; fc: fold change of DNA-modification level relative to the arrow-indicated condition; Pos. control: gDNA incubation with FPG and ENDOIV; Neg. control: dGTP incorporation instead of prop-dGTP.

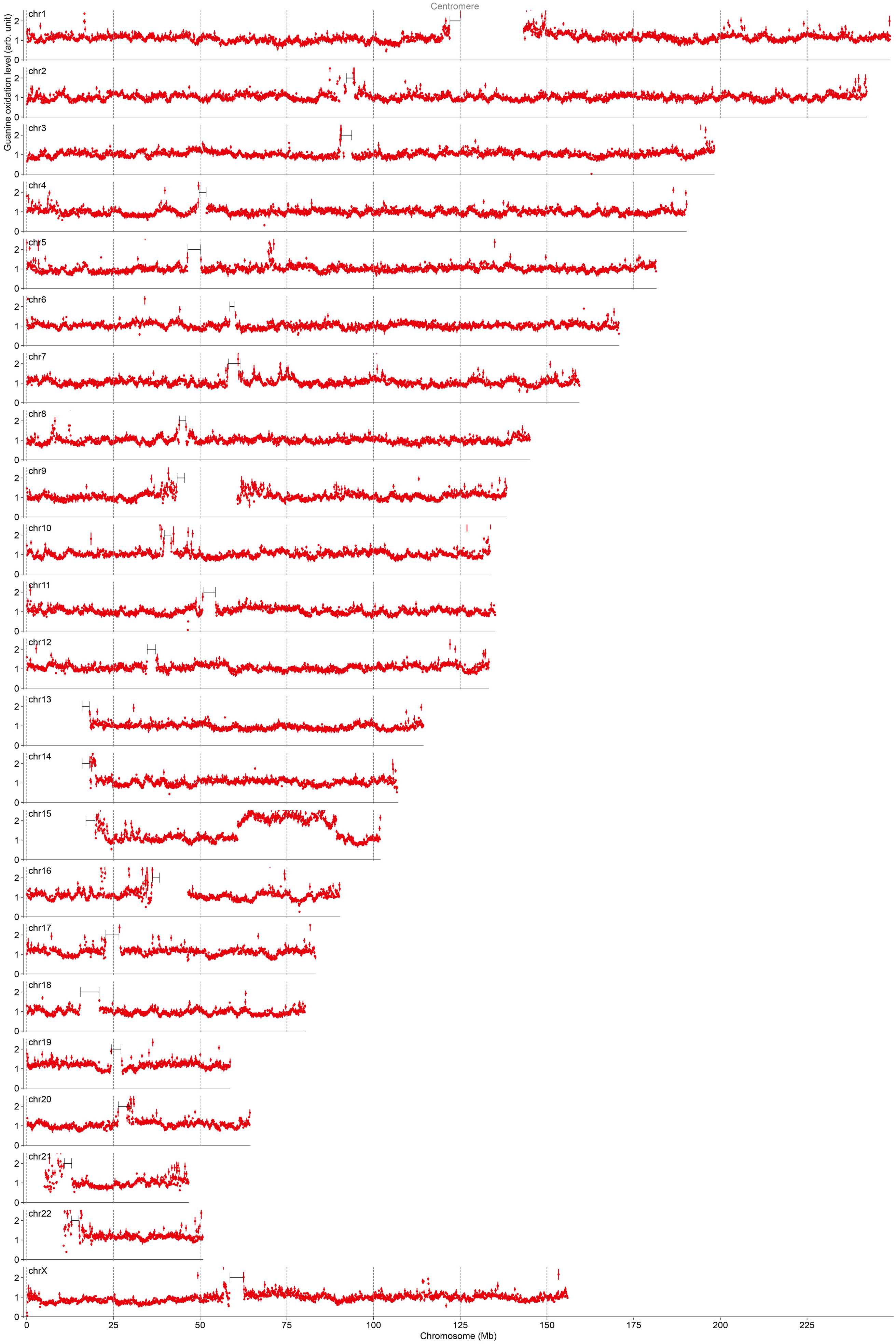

Centromere
Guanine oxidation level (arb. unit)
chr1
chr2
chr3
chr4
chr5
chr6
chr7
chr8
chr9
chr10
chr11
chr12
chr13
chr14
chr15
chr16
chr17
chr18
chr19
chr20
chr21
chr22
chrX
0
25
50
75
100
125
150
175
200
225
Chromosome (Mb)

<<< Supplementary Fig. 2. Genome-wide distribution of endogenous guanine oxidation levels at 100-Kb resolution in unexposed HAP1 cells. Shown: mean ± s.d. (marker and error bar) across n=3 biological replicates. Max Y-axis value: 99.5 percentile of the mean values. In detail, we isolated gDNA from HAP1 cells, treated it with ENDOIV to convert AP sites and phosphorylated breaks into 3'-OH breaks. These were ddNTP-blocked to conceal them from the oxidation map. Afterwards, we used FPG and ENDOIV to excise oxidized guanosines and Therminator IX to incorporate prop-dGTP into the resulting gaps. Only then was the DNA sonicated, end-repaired, and sequencing adapters were ligated. Next, we ligated biotinylated MoDIS to prop-dGTP sites via CuAAC, performed a streptavidin-based enrichment of DNA fragments ending at the original DNA-modification sites, PCR-amplified and sequenced the enriched fragments. To further minimize mapping of artefactual DNA oxidation, we used the antioxidants deferoxamine and n-tert-butyl-α-phenylnitrone in a buffer for cell-pellet resuspension, and 8-hydroxyquinoline in all DNA handling activities starting from gDNA isolation to labeling of guanine-oxidation sites with prop-dGTP.

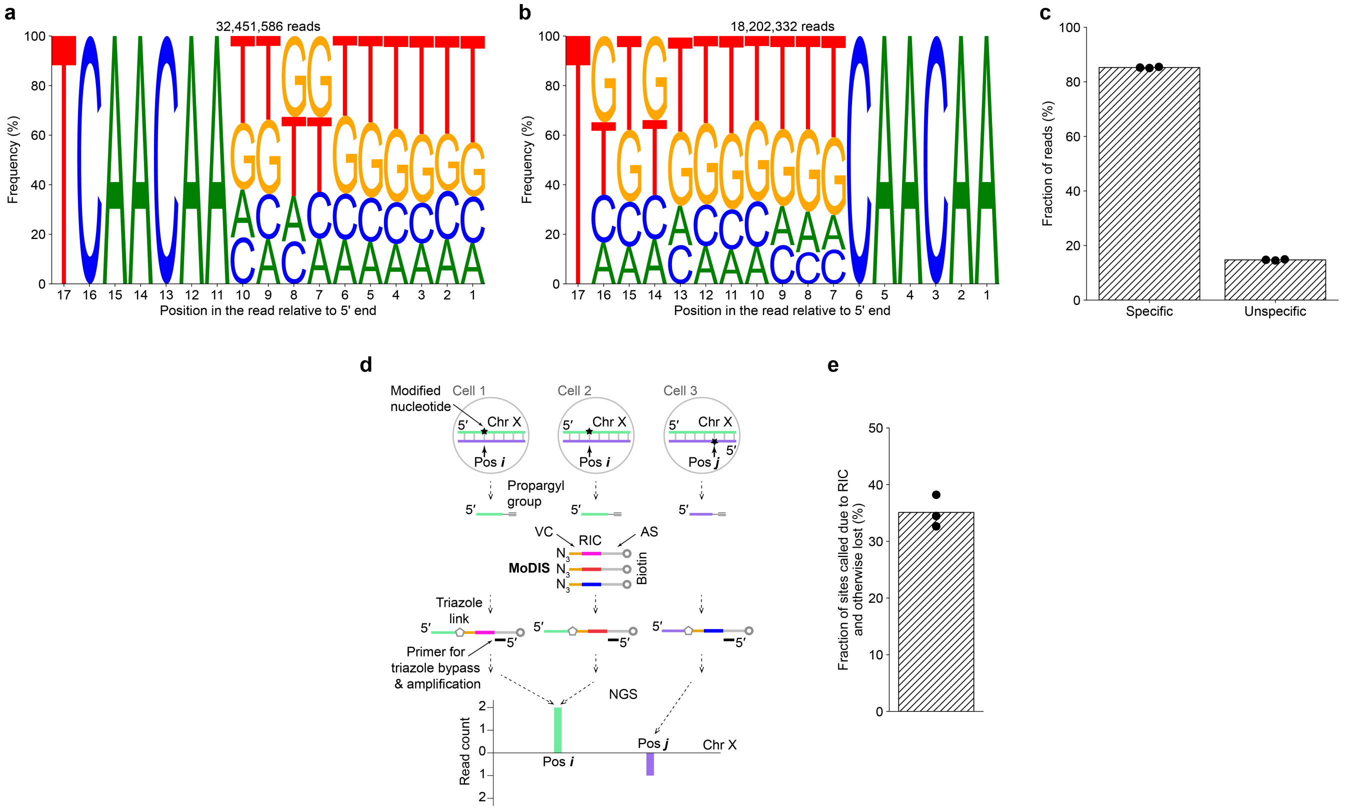

Supplementary Fig. 3. Increasing the specificity and sensitivity of click-code-seq with the upgraded design of the code adapter MoDIS that enables filtering out amplification artifacts and retaining identical reads originating from different cells/genomes. **a-b** VC (conserved positions) and RIC (variable positions) in the reads originating from MoDIS. The read sequences were complemented. Data: one replicate of endogenous-guanine-oxidation mapping in unexposed HAP1 cells is shown. MoDIS design, including VC and RIC, is provided in Extended Data Fig. 2a. **c** The fractions of specific, *i.e.*, MoDIS-containing reads (with VC, see **a-b**), and unspecific reads lacking VC. The reads in **a-b** correspond to one circular marker in the group of specific reads. Due to using MoDIS, the unspecific, *i.e.*, artefactual reads (~15%), can be identified and removed. **d** Schematic explanation of how RIC helps retain identical reads originating from different cells/genomes. **e** The usage of RIC in MoDIS elevates the number of identified oxidation sites by around 35%. The fraction relates the number of specific (*i.e.*, VC-containing) deduplicated reads with RIC sequences and the number of reads after computational removal of RIC sequences and deduplication. In panels **c,e**, n=3 biological replicates (circular markers) and their means (bars) of endogenous-oxidized-guanine mapping in unexposed HAP1 cells are shown.

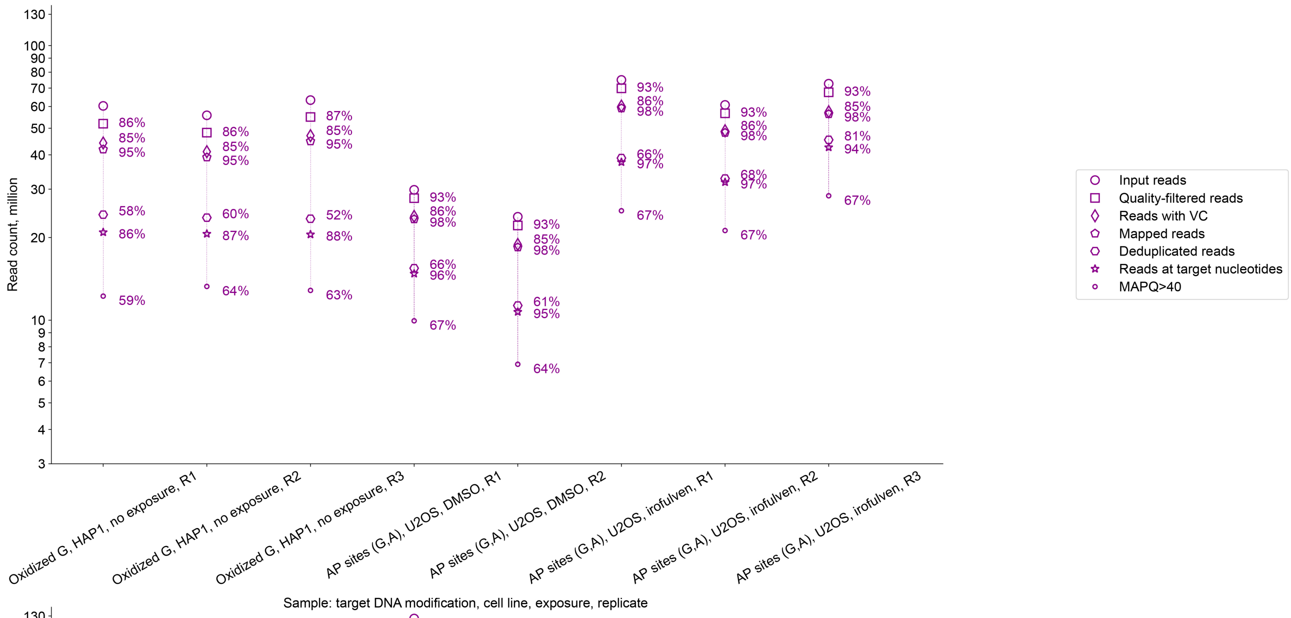

Read count, million
Input reads
Quality-filtered reads
Reads with VC
Mapped reads
Deduplicated reads
Reads at target nucleotides
MAPQ>40
Oxidized G, HAP1, no exposure, R1
Oxidized G, HAP1, no exposure, R2
Oxidized G, HAP1, no exposure, R3
AP sites (G,A), U2OS, DMSO, R1
AP sites (G,A), U2OS, DMSO, R2
AP sites (G,A), U2OS, irofulven, R1
AP sites (G,A), U2OS, irofulven, R2
AP sites (G,A), U2OS, irofulven, R3
Sample: target DNA modification, cell line, exposure, replicate

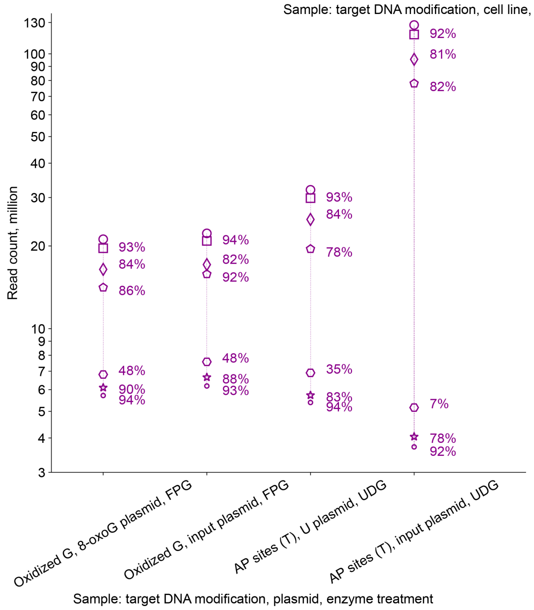

Read count, million
Oxidized G, 8-oxoG plasmid, FPG
Oxidized G, input plasmid, FPG
AP sites (T), U plasmid, UDG
AP sites (T), input plasmid, UDG
Sample: target DNA modification, plasmid, enzyme treatment

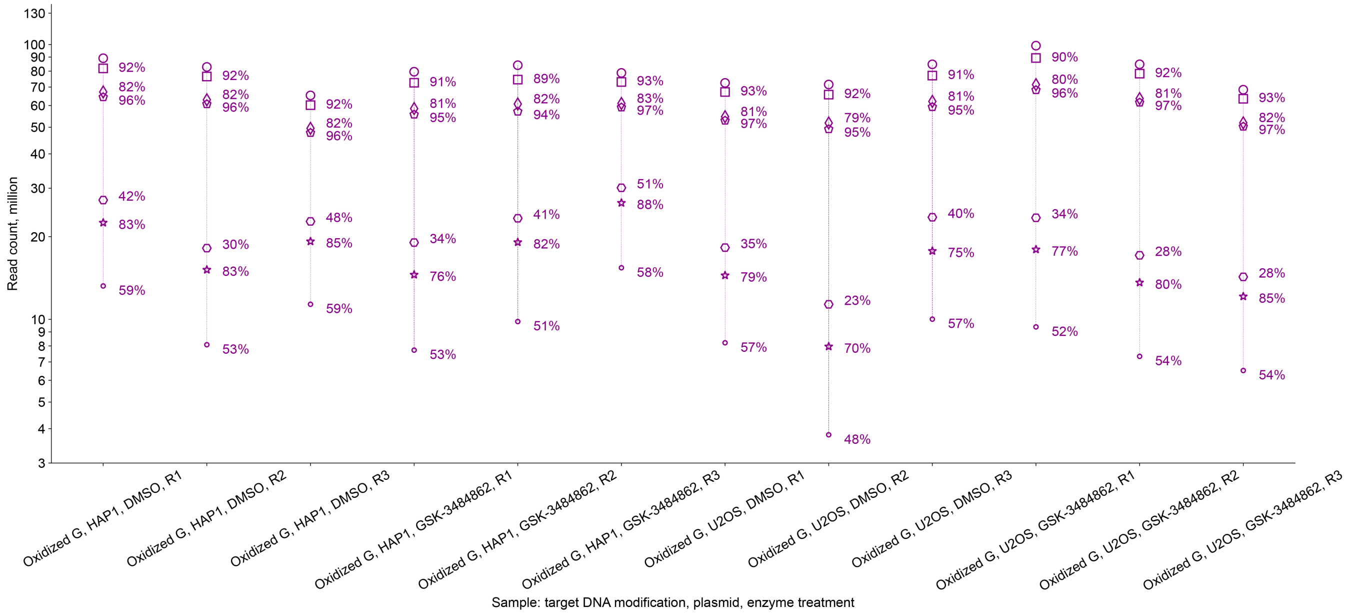

Read count, million
Oxidized G, HAP1, DMSO, R1
Oxidized G, HAP1, DMSO, R2
Oxidized G, HAP1, DMSO, R3
Oxidized G, HAP1, GSK-3484862, R1
Oxidized G, HAP1, GSK-3484862, R2
Oxidized G, HAP1, GSK-3484862, R3
Oxidized G, U2OS, DMSO, R1
Oxidized G, U2OS, DMSO, R2
Oxidized G, U2OS, DMSO, R3
Oxidized G, U2OS, GSK-3484862, R1
Oxidized G, U2OS, GSK-3484862, R2
Oxidized G, U2OS, GSK-3484862, R3
Sample: target DNA modification, plasmid, enzyme treatment

<<< Supplementary Fig. 4. Read counts throughout consecutive preprocessing steps in all samples generated in this work. VC: validation code (Extended Data Fig. 2a), reads at target nucleotides: reads calling DNA modification for the expected nucleotide (G for oxidized G, A and G or T for AP sites); MAPQ>40: reads with high mapping quality (probability of wrong alignment of the read to the template genome is smaller than 10^{-4}).

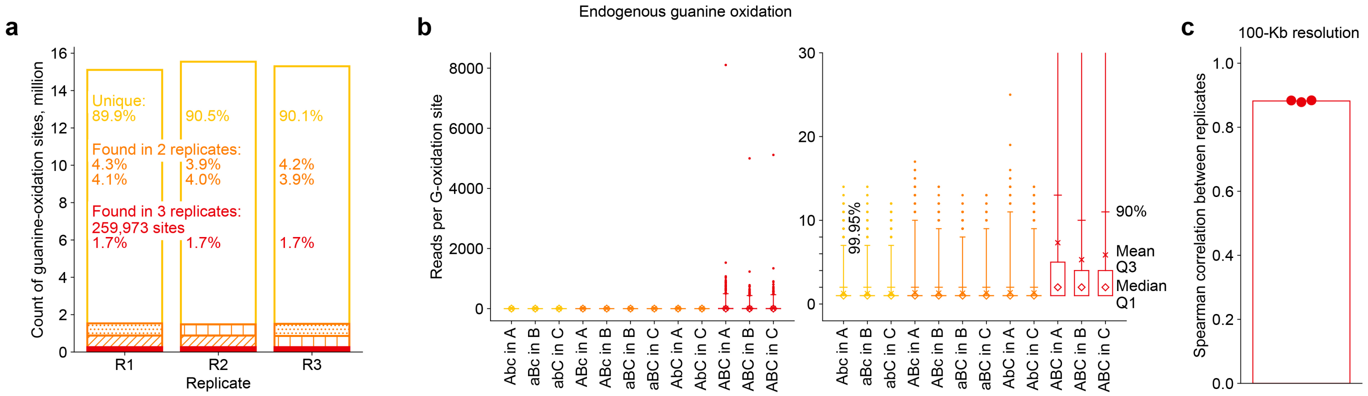

Supplementary Fig. 5. Reproducibility of DNA-oxidation signals at the single-nucleotide level and at 100-Kbp resolution. a Counts of single-nucleotide sites of endogenous guanine oxidation in HAP1 cells after categorizing the sites into replicate-specific and reproducible across replicate experiments. **b** The distribution of the number of unique reads (with individual RICs) supporting the DNA modification sites across the replicate experiments and the categories of modification-site reproducibility. A, replicate R1; B, replicate R2; C, replicate R3; Abc, the DNA modification sites specific to replicate A (R1); ABc, the DNA modification sites found in replicates A (R1) and B (R2); ABC, the DNA modification sites found in three replicates. The box plot elements are denoted in the right plot. The reproducibly detected sites were identified by more reads with individual RICs (mean: 5.3-7.3 across replicates, 90th percentile: 10-13) compared to replicate-specific sites (mean: 1.3 across replicates, 90th percentile: 2), reflecting that more cells had an oxidized guanine at a reproducibly detected site compared to a replicate-specific site. **c** Pair-wise correlations (markers) among n=3 replicates for the mapping of endogenous guanine oxidation in HAP1 cells at 100-Kbp resolution. Bars: mean pair-wise correlations.

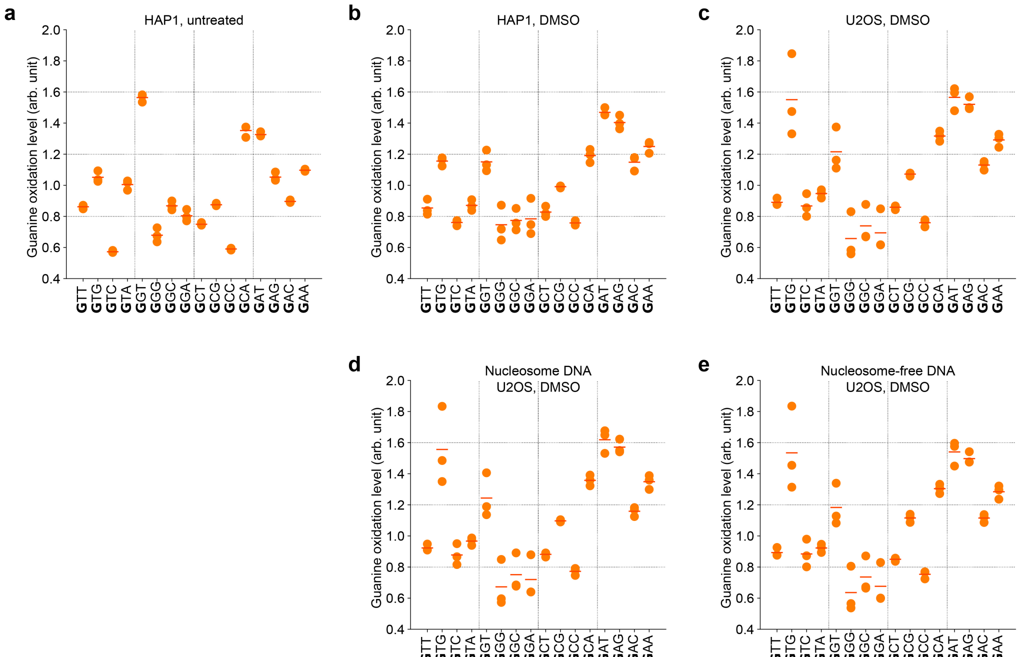

Supplementary Fig. 6. Oxidation level of 5′ guanine in trinucleotide contexts. Oxidation level of 5′ guanine (bold) in all nuclear-genome trinucleotide contexts in unexposed HAP1 cells (**a**), HAP1 cells exposed to DMSO (**b**), U2OS cells exposed to DMSO (**c**) as well as in trinucleotide contexts in nucleosome DNA (**d**) and nucleosome-free DNA (**e**) in U2OS cells exposed to DMSO. Unexposed HAP1 cells (**a**) were cultured continuously at 3% oxygen for 3 days, whereas DMSO-exposed HAP1 and U2OS cells were cultured at 3% oxygen for 3 days with 2 refreshments of media every 24 hours associated with exposure to atmospheric oxygen. The guanine oxidation levels are normalized by the count of respective trinucleotides in the reference genome. Circular markers: n=3 replicates, horizontal dashes: means.

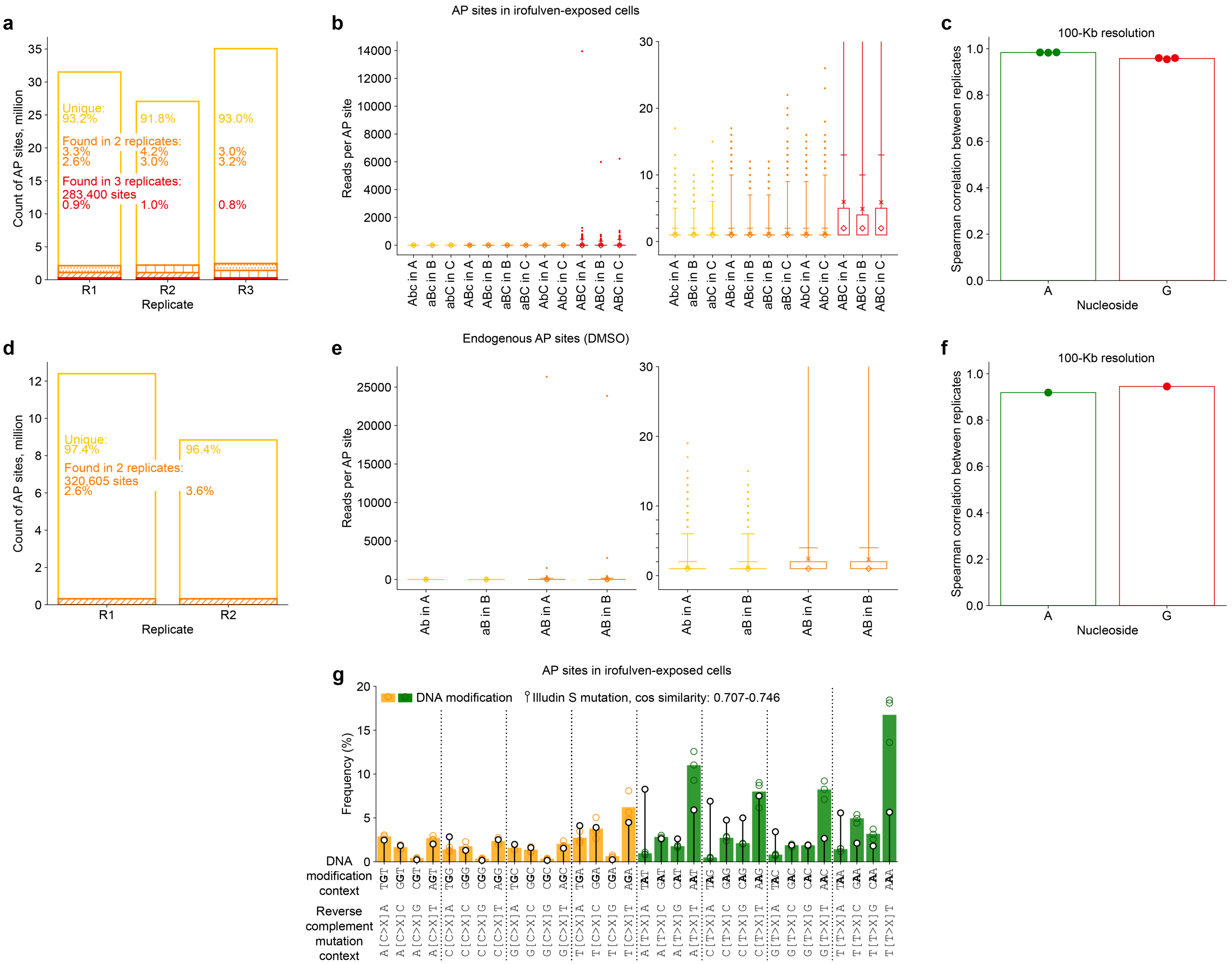

Supplementary Fig. 7. Reproducibility of AP-site signals at the single-nucleotide level and at 100-Kbp resolution, and irofulven-induced AP-site profile versus illudin S mutational signature. a,d Counts of single-nucleotide sites of depurination in irofulven-exposed U2OS cells (**a**) and in vehicle-(DMSO)-exposed U2OS cells (**d**) after categorizing the sites into replicate-specific and reproducible across replicate experiments. **b,e** The distribution of the number of unique reads (with individual RICs) supporting the DNA modification sites across the replicate experiments and the categories of modification-site reproducibility. A, replicate R1; B, replicate R2; C, replicate R3; Abc, the DNA modification sites specific to replicate A (R1); ABc, the DNA modification sites found in replicates A (R1) and B (R2); ABC, the DNA modification sites found in three replicates. The box plot elements are denoted in Supplementary Fig. 5b. **c,f** Pair-wise correlations (markers) among n=3 (**c**) or n=2 (**f**) replicates for the mapping of AP sites in irofulven-exposed U2OS cells (**c**) and in vehicle-(DMSO)-exposed U2OS cells (**f**) at 100-Kbp resolution; A: adenosine-derived, G: guanosine-derived AP sites. Bars: respective mean pair-wise correlations. **g** The alignment between Illudin S mutational signature and the distribution of AP sites regarding trinucleotide context in U2OS cells exposed to irofulven (n=3). The plot is built analogously to Fig. 2g. Irofulven-induced alkylation sites in extracted gDNA were allowed to convert to AP sites (37 °C, 18 h; spontaneous depurination half-life is in the range of 2-8.5 h[1]). We blocked DNA breaks by adding ddNTP with Therminator IX, converted all AP sites, whether drug-induced or endogenous, to gaps using ENDOIV. Next, we incorporated prop-dGTP and prop-dATP in the gaps, followed by further click-code-seq steps, the same as for oxidation mapping. By the identical procedure, we mapped endogenous AP sites in vehicle (0.03% DMSO) exposed cells.

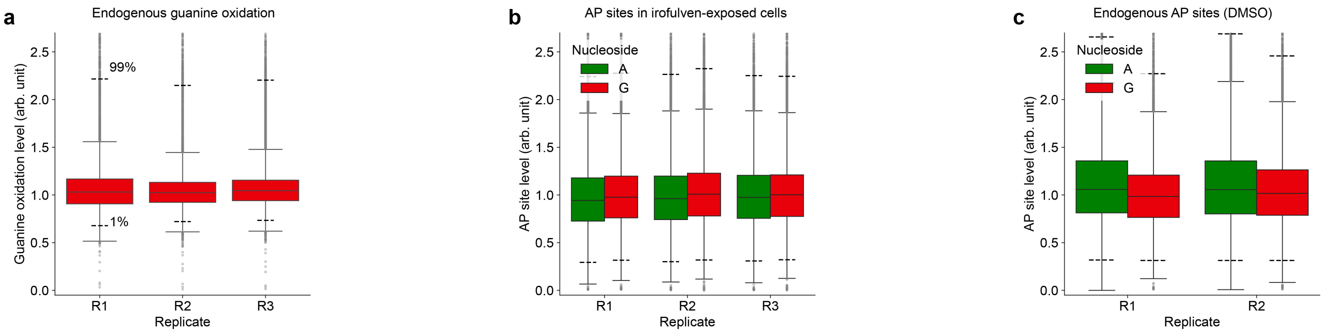

Supplementary Fig. 8. Distribution of DNA modifications at 100-Kbp resolution. The 100-Kbp-bin distributions of endogenous guanine oxidation in HAP1 cells (**a**, the same data as in Supplementary Fig. 2), AP sites in irofulven-exposed U2OS cells (**b**) and AP sites in vehicle-(DMSO)-exposed U2OS cells (**c**) in individual replicates; A: deoxyadenosine-derived, G: deoxyguanosine-derived AP sites. Dashed lines: 1st and 99th percentiles. Boxes are interquartile ranges, internal horizontal lines are medians, whiskers extend to the furthest datapoint within 1.5x interquartile range, datapoints beyond are shown as small markers.

Supplementary Table 1. DNA oligonucleotides. 5'–3' orientation; oxoG, 8-oxoG; THF, tetrahydrofuran; Phos, phosphorylated; U, uracil; *, phosphothioester linkage; N_3, azido; TEG, tetra-ethylene-glycol linker; N, randomized position.

Name	Sequence
IL1-T30	ATCTTTTGTAGCATACATTGAAGATGTGGC
IL-1	GCCACATCTTCAATGTATGCTACAAAAGAT
IL-1_oxoG	GCCACATCTTCAAT***oxoG***TATGCTACAAAAGAT
IL-1_THF	GCCACATCTTCAATGT***THF***TGCTACAAAAGAT
For_oxoG	**Phos**-AGCTTTGTCCTCTGCTTGCG
Rev_oxoG	**Phos**- GATCCGCAAGCA***oxoG***AGGACAA
For_U	**Phos**-AGCTTTGTCCTATGCTTGCG
Rev_U	**Phos**- GATCCGCAAGCA***U***AGGACAA
For_pEGFP	GGAGGTCTATATAAGCAGAGCTGG
NEB_P7	AGACGTGTGCTCTTCCGATCTAGAAGGCCTAG*****T
NEB_P5	**Phos**-CTAGGCCTTCTAAGGAGATGTTGATGTGCTGC
MoDIS_1	**N_3**-TNNNNNNNNNNCAACAAAGATCGGAAGAGCGTCGTGTAGG-***TEG***-Biotin
MoDIS_2	**N_3**-TCAACAANNNNNNNNNNAGATCGGAAGAGCGTCGTGTAGG-***TEG***-Biotin
Pr-P7	AGACGTGTGCTCTTCCGATCTA
exUMI	CCTACACGACGCTCTTCCGATC
i5/i7 indexing primers	These sequences are from the NEBNext® Multiplex Oligos for Illumina® (Index Primers Set 1) (NEB E7335S) and can be found in the respective product manual.

Supplementary Note 1. Quantification of modifications in genomic DNA via click-fluoro-quant

We used click-fluoro-quant to distinguish and measure oxidized bases, AP sites and breaks in human genomic DNA (gDNA), by employing corresponding enzymes to convert each modification selectively into 3′-OH groups for subsequent fluorescent labeling (Fig. 1a). When gDNA from the human chronic myeloid leukemia cell line HAP1 was treated with T4 PNK to cleave 3′-phosphorylated DNA breaks, we observed a 1.6-fold increase in fluorescence compared to untreated gDNA where only pre-existing 3′-OH-ended DNA breaks and termini were labeled (Fig. 1b). When the gDNA was treated with ENDOIV, which has both AP-lyase and phosphatase activities[2], we observed a 2.8-fold increase in fluorescence compared to untreated gDNA and a 1.8-fold increase compared to the T4 PNK-treated samples. The difference between the fluorescence increases for ENDOIV vs. T4 PNK treatments reflects AP sites, accounting for 44% of the signal in ENDOIV-treated gDNA (Fig. 1b). When gDNA was treated with combination of FPG and ENDOIV, which together recognize oxidized bases, AP sites and phosphorylated breaks, we observed an over-4-fold increase in fluorescence compared to when there was no enzymatic treatment (Fig. 1b). This fluorescence signal was 1.6-fold higher than when gDNA was treated with combination of FPG and T4 PNK (Fig. 1b), suggesting that ENDOIV more efficiently cleaved the AP sites produced by FPG.

After cell exposure to potassium bromate, there was no observed change in the levels of breaks or AP sites (Fig. 1c, no enzyme or ENDOIV treatment), however, we observed a 1.8-fold increase of oxidized sites (Fig. 1c, signal differential between ENDOIV+FPG vs. ENDOIV treatments), consistent with a previous report[3] and quantification of 8-oxoG under the same conditions by HPLC-MS/MS (2.2-fold increase, Fig. 1d). Next, immortalized human skin BJ-5ta cells were irradiated with a dose of 10 J/cm^2 UVA, leading to a 1.3-fold increase in breaks plus AP sites (Fig. 1e, ENDOIV), and a 1.2-fold increase in DNA oxidation (Fig. 1e, differential between ENDOIV+FPG vs. ENDOIV). Finally, we used the chemotherapeutic drug irofulven[1,4] to induce depurination in gDNA of human osteosarcoma-derived U2OS cells and identified a drug-concentration-dependent increase in the abundance of induced AP sites (Fig. 1f) but not breaks (Extended Data Fig. 1d). These data support the versatility of using click-fluoro-quant to determine alterations in the targeted modifications induced by chemical or radiation exposure.

For genome-wide mapping of oxidized guanines, both FPG and ENDOIV are needed to generate 3′-OH groups as a marker of the oxidized-guanine site, however, 3′-OH groups exist independently. Furthermore, 3′-OH groups are created by the action of ENDOIV on pre-existing endogenous AP sites and phosphorylated breaks, and we wish to exclude these for accurate mapping of oxidized guanines. Therefore, we established a workflow that involved first ENDOIV treatment creating unspecific 3′-OH groups and afterwards the removal of the breaks by either repairing them with deoxynucleotide addition and ligation[5] (Fig. 1g, repair) or blocking them by adding non-reactive dideoxynucleotides (ddNTP)[6] to the sites (Fig. 1g, blocking). There were slightly fewer unspecific 3′-OH groups after blocking vs repair (Fig. 1g), even after attempting to optimize the latter as was done previously[7] (Extended Data Fig. 1e), so we adopted the blocking strategy for sequencing.

Finally, to address artefactual modifications, we evaluated their levels associated with gDNA-sample-handling factors (Supplementary Fig. 1), which suggested avoiding heat inactivation of enzymes. In addition, we identified that the ddNTP blocking excluded most 3′-OH groups generated by sonication (Fig. 1h, sonication+blocking vs. sonication). Sonication is used to fragment gDNA to prepare libraries for

sequencing and is established as a major source for artifactual DNA modifications[8]. However, ddNTP-blocked non-sonicated gDNA still had almost 4-fold less unspecific 3′-OH groups than blocked sonicated samples (Fig. 1h, blocking vs. sonication+blocking). Thus, in order to prepare sequencing libraries, fragmentation by sonication should be performed after both ddNTP blocking of background 3′-OH groups and labeling of the target DNA modifications.

Supplementary Note 2. 5′-guanine is not preferentially oxidized in 5′-GG-3′ context in the human genome

Oxidative DNA modifications can be induced at a distance – potentially over kilobase lengths – through electron hole transport along the π-stacked base pairs of B-DNA. This process preferentially targets the 5′-guanine in guanine doublets and triplets[9,10], as the adjacent downstream guanine lowers the ionization potential of the 5′-guanine[11,12], making it more susceptible to oxidation. One way such electron holes can be generated is through the attack of the carbonate radical anion ($CO_3^{\bullet-}$)[13], a likely product of the intracellular Fenton reaction[14,15]. To search for indications of at-a-distance oxidation, we compared the oxidation levels of 5′-guanine across all triplets 5′-**G**XY-3′ in unexposed HAP1 cells as well as DMSO-exposed HAP1 and U2OS cells (Supplementary Fig. 6a-c). With the exception of the triplet **G**GT, triplets **G**GG, **G**GC and **G**GA showed some of the lowest oxidation levels of 5′-guanine in each cell culture condition, which opposes the expectation from oxidation induced at a distance. Since DNA wrapped around nucleosomes deviates from canonical B-DNA by exhibiting compressed minor grooves, expanded major grooves and variable helical parameters[16], we conjectured that nucleosome-free DNA would have stronger indication of oxidation induced at a distance. Yet, using available MNase-seq mapping of nucleosomes in U2OS cells[17], we did not observe higher oxidation levels of 5′-guanine in 5′-**G**GY-3′ triplets in nucleosome-free DNA compared to nucleosome DNA (Supplementary Fig. 6d-e). In fact, nucleosome DNA showed slightly higher guanine oxidation levels in 5′-**G**AY-3′ triplets (Supplementary Fig. 6d-e). Overall, the absence of preferential 5′-guanine oxidation in guanine doublets may suggest either the repair of oxidation events occurring at a distance or the predominance of oxidation mechanisms that do not involve hole generation, such as those mediated by hydroxyl radicals[15].

Supplementary Note 3. DNA-modification levels in the context of replication

Using available replication timing data (Repli-Seq) for HAP1[18] and U2OS[19] cell lines, we found that the levels of guanine oxidation and depurination are higher in earlier replicated DNA regions (Fig. 3b,e,h), which correspond to open chromatin[20]. We could also infer replication fork directionality in certain large genomic regions (> 200 Kb) where Repli-Seq signal steadily decreases or increases (timing transition regions[21]) and hence measure the burden of DNA modifications on respective leading and lagging strand templates. Here, we identified that endogenous guanine oxidation level has a replicative strand bias such that 55.3-58.9% of 50 Kbp bins in the analyzed regions reveal higher guanine oxidation level on the lagging strand template (Fig. 3c), which may reflect that it is more single-stranded than the leading strand template[22] and thus more prone to endogenous guanine oxidation. While AP sites at adenines in irofulven-exposed cells did not show a replicative strand bias consistent across separate experiments (Fig. 3f), endogenous depurination level was found higher in the lagging strand template versus the leading strand template in more (53.3-55.1%) of analyzed bins across independent measurements (Fig. 3i). Overall, guanine oxidation and AP sites are associated more with the genomic regions of active chromatin and early replication, and endogenous DNA modifications have a weak replicative strand bias towards the lagging strand template.

Supplementary Note 4. Guanine-oxidation strand bias in trinucleotide contexts within gene bodies.

To confirm that the observed dependence of guanine oxidation on strand assignment and gene-expression level (Fig. 4a-b) is not due merely to confounders like varying sequence composition throughout gene expression tiers or different guanine oxidation propensities across trinucleotides (Fig. 2b), we analyzed the same relationship on the level of individual trinucleotides (Extended Data Fig. 4a). Here, we found that the median guanine-oxidation level changed with gene expression in 15 out of 16 trinucleotides. In 12 cases, we observed generally increasing dependencies with different maximal fold changes compared to the unexpressed-gene tier, namely, around 15.5-29.5% in AGA, TGN, AGG, GGA, AGC, 38.2% in GGG, 50.1% in AGT, 58.9-60.3% in GGC, GGT for the non-transcribed strand (Extended Data Fig. 4a). In CpG dimers CGG, CGC and CGA, median guanine-oxidation levels were zero in unexpressed and lowly expressed genes, then increased to a peak at around the 60% gene-expression tier and dropped to zero again in the most expressed genes (Extended Data Fig. 4a). Interestingly, we identified that the extent of the guanine-oxidation strand bias also varies across the trinucleotides, being most pronounced in GGT, GGA, TGT (17.6-21.6%), virtually absent in AGC, CpG dimers CGN, and intermediate in the rest (Extended Data Fig. 4a). Thus, the overall guanine-oxidation level and its strand bias increase as function of gene expression, with most individual trinucleotides within genes showing the same pattern, however, to varying degrees.

Supplementary Note 5. AP-site strand bias in trinucleotide contexts within gene bodies.

Analyzing median AP-site levels in individual trinucleotide contexts (Extended Data Fig. 6 and 7), we observed elevating DNA-modification burdens with increasing gene expression for almost all trinucleotides for both irofulven-induced and endogenous depurination. Yet, the trinucleotides were found to vary in the maximal fold changes, spanning, in case of irofulven-induced damage, from 39.4% for in AAA up to infinity (elevation from zero) in TAN, CAT (Extended Data Fig. 6a) and CGN (Extended Data Fig. 7a) in the non-transcribed strand. Similarly, the strand biases proved to vary across the trinucleotides from no pronounced strand differences of irofulven-induced depurination in CAN (Extended Data Fig. 6a) and CGN (Extended Data Fig. 7a) along the gene-expression tiers to over 30% difference between the non-transcribed and transcribed strands for GAC and TAT in the tier of the most expressed genes (Extended Data Fig. 6a). Supporting that the endogenous adenosine-derived AP sites do not confound the mapping of irofulven-induced ones, we noticed different gene-expression-dependent patterns at individual trinucleotides in vehicle-treated samples compared to irofulven exposure (Extended Data Fig. 6b vs 6a), with fewer trinucleotides showing the strand bias and with generally higher fold changes as the background adenine depurination is mostly zero in unexpressed genes (Extended Data Fig. 6b). For example, in AAA context, there is a pronounced strand bias under irofulven exposure (Extended Data Fig. 6a), while vehicle exposure is associated with no difference between the gene strands (Extended Data Fig. 6b), which may be explained by polymerase stalling at irofulven-adenosine adduct in the first case and a lack of polymerase stalling at the AP site in the second case. On the contrary, trinucleotide-specific guanosine-derived AP-site levels were more similar between irofulven- and vehicle-exposed samples (Extended Data Fig. 7a-b), which, given lower rate of guanosine modification by irofulven (Fig. 2e), could potentially reflect a higher confounding effect of endogenous guanosine depurination for mapping this DNA modification in irofulven-exposed samples.

Supplementary Note 6. DNA oxidation and depurination diverge in the proximity of transcription start site.

Besides the observations of strand bias in the gene bodies, another prominent feature of the DNA-modification profiles with respect to gene regions is a signal deviation in the proximity of TSS. For example, close to TSS, there are a sharp drop of guanine oxidation (Fig. 4c) (which has been disputed in literature with both depletion[6,23,24] and enrichment observations[25-27]) and a spike of irofulven-induced adenosine depurination (Fig. 4g). To further explore these features, we measured guanine-oxidation level in the signal drop between -400 and +600 bases around TSS (vertical dashes in Fig. 4c) and related these values to gene expression. Surprisingly, we discovered a non-monotonic relationship (Extended Data Fig. 8a-b) in which guanine oxidation is highest in unexpressed genes, decreases and reaches a minimum (25.4-29.2% reduction) in the 50% and 60% tiers of gene expression, but then increases again (by around 23%). Nonetheless, guanine-oxidation levels in highly expressed genes were still lower than in unexpressed genes (Extended Data Fig. 8a-b). The levels of adenosine-derived AP sites, both irofulven-induced and endogenous, in the proximity of TSS (-1000 and 0 bases, vertical dashes in Fig. 4g and Extended Data Fig. 5g) had a different behavior (Extended Data Fig. 8c-f), almost always increasing as a function gene expression with a maximal fold change of 45%-46.8% (Extended Data Fig. 8d,f) and different from the patterns of guanosine depurination (Extended Data Fig. 8g-j). Thus, burdens of DNA oxidation and alkylation-induced and endogenous depurination within the promoter regions of genes vary in relation to gene expression level, however, the respective associations have different forms. It is known that 8-oxoG in promoter regions can regulate gene expression via BER and altered G-quadruplex structures, as seen for *VEGF* and *NTHL1*[28,29]. Additionally, a recent genome-wide analysis reported reduced 8-oxoG in G-quadruplex sites but increased 8-oxoG in non-G-quadruplex potential-G-quadruplex sequences[23]. Our observations of varying levels of adenosine depurination and guanine oxidation in promoters as a function of gene expression may indicate a contribution of these modifications or their repair intermediates to transcriptional regulation.

Supplementary Note 7. Analysis of DNA modifications in the D-loop region

Examining single-nucleotide coverage by reads with unique RICs, we revealed that guanine at position 16,103 (G16,103|**–**) and adenine at position 16,104 (A16,104|**–**) in the **–** strand had the highest modification coverage throughout mtDNA in respective mapping of oxidized guanines, deoxyguanosine- and deoxyadenosine-derived AP sites (Extended Data Fig. 9e-i). These single-nucleotide signals were the largest contributors to the D-loop-localized peaks observed in the bin analyses (Fig. 5a-d, Extended Data Fig. 9d). The D-loop region includes a third strand, 7S DNA, which represents a linear copy of the **–** strand of unknown function[30] with the 3′-end nucleotide predominantly at position 16,105 and less abundantly at positions 16,104 and 16,103[31]. The uncovered DNA-modification signals at G16,103|**–** and A16,104|**–** may thus be localized in either 7S DNA or in the circular **–** strand. The occurrence of such strong DNA-modification signals close to the 3′ end of 7S DNA may suggest their functional relevance in the control of 7S DNA synthesis termination and hence mtDNA replication[30,32]. Thus, mtDNA exhibits an asymmetric distribution of endogenous guanine oxidation and adenosine depurination and produces strong DNA-modification signals at two single-nucleotide positions in the D-loop region.

Supplementary Discussion 1. Comparison with click-code-seq v2.0.

In a recent adaptation of click-code-seq, termed click-code-seq v2.0, the method was used to map 8-oxoG at a known site in plasmid DNA[33]. This version reversed the click partners by utilizing commercially available 3′-azido-2′,3′-ddGTP and a 5′-alkynyl-modified code sequence to expand the method applications by eliminating the need for custom chemical synthesis done in the original protocol[6]. In our study, we retained the original click partner configuration, using *O*-3′-propargyl-2′,3′-ddNTPs and a 5′-azido-modified code sequence (MoDIS), as the propargyl-modified nucleotides are now commercially available. As in click-code-seq v2.0, we employed endonuclease IV (EndoIV) to remove the 3′ phosphate after Fpg-mediated 8-oxoG excision since EndoIV is significantly more efficient than APE1[33], which was used in the original protocol. While click-code-seq v2.0 utilized the Klenow fragment exo^{-} polymerase to incorporate the modified ddGTP into gaps generated by repair enzyme treatment of 8-oxoG, we followed the original protocol and used Therminator IX polymerase, however, both enzymes achieve near-quantitative yields[6,33].

References:

1. Gong, J.C. et al. Depurinating acylfulvene-DNA adducts: Characterizing cellular chemical reactions of a selective antitumor agent. *Journal of the American Chemical Society* **129**, 2101-2111 (2007).
2. Daley, J.M., Zakaria, C. & Ramotar, D. The endonuclease IV family of apurinic/apyrimidinic endonucleases. *Mutation Research/Reviews in Mutation Research* **705**, 217-227 (2010).
3. Kawanishi, S. & Murata, M. Mechanism of DNA damage induced by bromate differs from general types of oxidative stress. *Toxicology* **221**, 172-178 (2006).
4. Herzig, M.C.S., Arnett, B., MacDonald, J.R. & Woynarowski, J.M. Drug uptake and cellular targets of hydroxymethylacylfulvene (HMAF). *Biochemical Pharmacology* **58**, 217-225 (1999).
5. Zatopek, K.M. et al. RADAR-seq: A RAre DAmage and Repair sequencing method for detecting DNA damage on a genome-wide scale. *DNA Repair (Amst)* **80**, 36-44 (2019).
6. Wu, J., McKeague, M. & Sturla, S.J. Nucleotide-Resolution Genome-Wide Mapping of Oxidative DNA Damage by Click-Code-Seq. *J Am Chem Soc* **140**, 9783-9787 (2018).
7. Shu, X.T. et al. Genome-wide mapping reveals that deoxyuridine is enriched in the human centromeric DNA. *Nature Chemical Biology* **14**, 680–687 (2018).
8. Costello, M. et al. Discovery and characterization of artifactual mutations in deep coverage targeted capture sequencing data due to oxidative DNA damage during sample preparation. *Nucleic Acids Research* **41**, e67-e67 (2013).
9. Hall, D.B., Holmlin, R.E. & Barton, J.K. Oxidative DNA damage through long-range electron transfer. *Nature* **382**, 731-735 (1996).
10. Arnold, A.R., Grodick, M.A. & Barton, J.K. DNA Charge Transport: from Chemical Principles to the Cell. *Cell Chem Biol* **23**, 183-197 (2016).
11. Senthilkumar, K., Grozema, F.C., Guerra, C.F., Bickelhaupt, F.M. & Siebbeles, L.D.A. Mapping the Sites for Selective Oxidation of Guanines in DNA. *Journal of the American Chemical Society* **125**, 13658-13659 (2003).
12. Sugiyama, H. & Saito, I. Theoretical Studies of GG-Specific Photocleavage of DNA via Electron Transfer: Significant Lowering of Ionization Potential and 5′-Localization of HOMO of Stacked GG Bases in B-Form DNA. *Journal of the American Chemical Society* **118**, 7063-7068 (1996).
13. Rokhlenko, Y., Geacintov, N.E. & Shafirovich, V. Lifetimes and Reaction Pathways of Guanine Radical Cations and Neutral Guanine Radicals in an Oligonucleotide in Aqueous Solutions. *Journal of the American Chemical Society* **134**, 4955-4962 (2012).
14. Illés, E., Mizrahi, A., Marks, V. & Meyerstein, D. Carbonate-radical-anions, and not hydroxyl radicals, are the products of the Fenton reaction in neutral solutions containing bicarbonate. *Free Radical Biology and Medicine* **131**, 1-6 (2019).
15. Fleming, A.M. & Burrows, C.J. Chemistry of ROS-mediated oxidation to the guanine base in DNA and its biological consequences. *Int J Radiat Biol* **98**, 452-460 (2022).
16. Richmond, T.J. & Davey, C.A. The structure of DNA in the nucleosome core. *Nature* **423**, 145-150 (2003).
17. Devaiah, B.N. et al. BRD4 is a histone acetyltransferase that evicts nucleosomes from chromatin. *Nat Struct Mol Biol* **23**, 540-8 (2016).
18. Klein, K.N. et al. Replication timing maintains the global epigenetic state in human cells. *Science* **372**, 371-378 (2021).
19. Rivera-Mulia, J.C., Trevilla-Garcia, C. & Martinez-Cifuentes, S. Optimized Repli-seq: improved DNA replication timing analysis by next-generation sequencing. *Chromosome Research* **30**, 401-414 (2022).
20. Fu, H., Baris, A. & Aladjem, M.I. Replication timing and nuclear structure. *Curr Opin Cell Biol* **52**, 43-50 (2018).

21. Zhao, P.A., Sasaki, T. & Gilbert, D.M. High-resolution Repli-Seq defines the temporal choreography of initiation, elongation and termination of replication in mammalian cells. *Genome Biology* **21**, 76 (2020).
22. Hoopes, J.I. et al. APOBEC3A and APOBEC3B Preferentially Deaminate the Lagging Strand Template during DNA Replication. *Cell Rep* **14**, 1273-1282 (2016).
23. An, J. et al. Genome-wide analysis of 8-oxo-7,8-dihydro-2'-deoxyguanosine at single-nucleotide resolution unveils reduced occurrence of oxidative damage at G-quadruplex sites. *Nucleic Acids Res* **49**, 12252-12267 (2021).
24. Poetsch, A.R., Boulton, S.J. & Luscombe, N.M. Genomic landscape of oxidative DNA damage and repair reveals regioselective protection from mutagenesis. *Genome Biology* **19**, 215 (2018).
25. Ding, Y., Fleming, A.M. & Burrows, C.J. Sequencing the Mouse Genome for the Oxidatively Modified Base 8-Oxo-7,8-dihydroguanine by OG-Seq. *J Am Chem Soc* **139**, 2569-2572 (2017).
26. Park, J.W. et al. 8-OxoG in GC-rich Sp1 binding sites enhances gene transcription in adipose tissue of juvenile mice. *Scientific Reports* **9**, 15618 (2019).
27. Amente, S. et al. Genome-wide mapping of 8-oxo-7,8-dihydro-2'-deoxyguanosine reveals accumulation of oxidatively-generated damage at DNA replication origins within transcribed long genes of mammalian cells. *Nucleic Acids Res* **47**, 221-236 (2019).
28. Fleming, A.M., Ding, Y. & Burrows, C.J. Oxidative DNA damage is epigenetic by regulating gene transcription via base excision repair. *Proc Natl Acad Sci U S A* **114**, 2604-2609 (2017).
29. Fleming, A.M., Zhu, J., Ding, Y. & Burrows, C.J. 8-Oxo-7,8-dihydroguanine in the Context of a Gene Promoter G-Quadruplex Is an On–Off Switch for Transcription. *ACS Chemical Biology* **12**, 2417-2426 (2017).
30. Nicholls, T.J. & Minczuk, M. In D-loop: 40years of mitochondrial 7S DNA. *Experimental Gerontology* **56**, 175-181 (2014).
31. Doda, J.N., Wright, C.T. & Clayton, D.A. Elongation of displacement-loop strands in human and mouse mitochondrial DNA is arrested near specific template sequences. *Proceedings of the National Academy of Sciences* **78**, 6116-6120 (1981).
32. Bernardino Gomes, T.M., Vincent, A.E., Menger, K.E., Stewart, J.B. & Nicholls, T.J. Mechanisms and pathologies of human mitochondrial DNA replication and deletion formation. *Biochemical Journal* **481**, 683-715 (2024).
33. Xiao, S., Fleming, A.M. & Burrows, C.J. Sequencing for oxidative DNA damage at single-nucleotide resolution with click-code-seq v2.0. *Chemical Communications* **59**, 8997-9000 (2023).
