# Supplementary material for: Click-code-seq reveals strand biases of DNA oxidation and depurination in human genome
Source: Nat Chem Biol. 2025 Oct 31;22(5):716–27. doi: 10.1038/s41589-025-02052-6 (PMC13128490; doi:10.1038/s41589-025-02052-6)
Supplement: Supplementary file 2 — Reporting Summary [file 41589_2025_2052_MOESM2_ESM.pdf]

# nature portfolio

Corresponding author(s): Shana J. Sturla

Last updated by author(s): Aug 26, 2025

# Reporting Summary

Nature Portfolio wishes to improve the reproducibility of the work that we publish. This form provides structure for consistency and transparency in reporting. For further information on Nature Portfolio policies, see our Editorial Policies and the Editorial Policy Checklist.

## Statistics

For all statistical analyses, confirm that the following items are present in the figure legend, table legend, main text, or Methods section.

| n/a | Confirmed | |
|---|---|---|
| ☐ | ☒ | The exact sample size (*n*) for each experimental group/condition, given as a discrete number and unit of measurement |
| ☒ | ☐ | A statement on whether measurements were taken from distinct samples or whether the same sample was measured repeatedly |
| ☐ | ☒ | The statistical test(s) used AND whether they are one- or two-sided<br>*Only common tests should be described solely by name; describe more complex techniques in the Methods section.* |
| ☒ | ☐ | A description of all covariates tested |
| ☒ | ☐ | A description of any assumptions or corrections, such as tests of normality and adjustment for multiple comparisons |
| ☐ | ☒ | A full description of the statistical parameters including central tendency (e.g. means) or other basic estimates (e.g. regression coefficient) AND variation (e.g. standard deviation) or associated estimates of uncertainty (e.g. confidence intervals) |
| ☐ | ☒ | For null hypothesis testing, the test statistic (e.g. *F*, *t*, *r*) with confidence intervals, effect sizes, degrees of freedom and *P* value noted<br>*Give P values as exact values whenever suitable.* |
| ☒ | ☐ | For Bayesian analysis, information on the choice of priors and Markov chain Monte Carlo settings |
| ☒ | ☐ | For hierarchical and complex designs, identification of the appropriate level for tests and full reporting of outcomes |
| ☐ | ☒ | Estimates of effect sizes (e.g. Cohen's *d*, Pearson's *r*), indicating how they were calculated |

*Our web collection on statistics for biologists contains articles on many of the points above.*

## Software and code

Policy information about availability of computer code

**Data collection**

In click-fluro-quant, fluorescence was recorded with an Infinite Pro M200 Plate Reader (Tecan).
In click-code-seq, DNA libraries were sequenced on an Illumina NovaSeq 6000 with a single-read protocol and the read length of 101 bp (R1).
The liquid chromatography-nanoelectrospray ionization-tandem mass spectrometry (LC-NSI-MS/MS) was performed on a TSQ Quantiva triple quadrupole mass spectrometer (ThermoFisher Scientific, San Jose, CA, United States) coupled to an ACQUITY UPLC M-Class (Waters, Milford, MA, United States) system using nanoelectrospray ionization. The analysis was conducted using a capillary column (150 µm ID, 5.5 cm packing length, 15 µm orifice) created by hand filling a commercially available fused-silica emitter (MSWIL, Aarle-Rixtel, Noord-Brabant, Netherlands) with HSS T3 separation media (Waters, Milford, MA, United States). The quantitation of the analytes was done by using the mass spectrometers vendor software package Quan Browser in the software suite Xcalibur based on the peak areas and the constructed calibration curves.
DNA methylation profiling was performed via Infinium MethylationEPIC BeadChip v2.0 at Life & Brain Gmbh, Germany. Specifically, 1) Standard Illumina Protocol was used for labeling, 2) DNA was bisulphite converted with Zymo EZ-96 DNA Methylation-Lightning Kit (D5033), amplified, fragmented and hybridized to human Infinium MethylationEPIC BeadChip v2.0 (Illumina) using standard Illumina protocol, 3) arrays were imaged using iScan System and standard recommended Illumina scanning settings.

**Data analysis**

1) For all plots concerning click-fluoro-quant and related statistical analyses, we used Jupyter notebooks employing the modules numpy version 1.22.4, scipy version 1.8.1, pandas version 1.4.2, matplotlib version 3.5.2 and seaborn version 0.11.2 in Python version 3.10.4 [GCC 8.2.0]. The code: https://gitlab.ethz.ch/eth_toxlab/click-code-seq/, folder: click-fluoro-quant.
2) The custom code for analyzing click-code-seq data and plotting figures is available at https://gitlab.ethz.ch/eth_toxlab/click-code-seq. The quality of the raw sequencing data was checked via FastQC version 0.11.9 or 0.12.1 (GSK-3484862 experiment, GE; plasmids, P). Low-quality reads and adapter-containing reads were removed via trimmomatic version 0.38 or 0.39 (GE, P). We retained only those reads that contained a validation code (VC). The first 17 nucleotides corresponding to VC and randomized index code (RIC) were clipped from the read sequences

and appended to the read names via the tool extract of umi_tools toolkit version 1.1.2 or 1.1.4 (GE, P). In case of samples from cells, reads were mapped to human reference genome GRCh38 via bowtie2 version 2.3.5.1 or 2.5.1 (GE), using pre-built bowtie2 index from https://genome-idx.s3.amazonaws.com/bt/GRCh38_noalt_as.zip, and applying otherwise standard settings. In case of samples originating from plasmids, we built bowtie2 indexes for three plasmids, namely, i) input plasmid (vector), ii) plasmid with triple 8-oxoG insert, 8-oxoG represented by G, and iii) plasmid with triple U insert, U represented by T (triple insert explained in Extended Data Fig. 2b); the plasmid sequences were flanked by 100 nucleotides to mimic circular structure in read alignment; these indexes were used to map reads via bowtie2 2.5.1. Read duplicates were removed by the tool dedup of umi_tools toolkit version 1.1.2 or 1.1.4 (GE, P), grouping reads with the same code sequence stored in the read name (method=unique). Samtools version 1.12 or 1.17 (GE, P) were employed to sort, index and generate statistics of bam files. bedtools2 version 2.29.2 or 2.31.0 (GE, P) were used to retrieve the coordinates of mapped and deduplicated reads and extract the sequence context of DNA modifications from the reference genome. Each read represented one unit of DNA-modification signal, which we positioned at the nucleotide of the 5′ end of the read. Since a read was the reverse complement of the DNA fragment captured in the method employing MoDIS, the strand of the nucleotide bearing the signal was changed to the opposite one. In GE, we retained reads with mapping quality (MAPQ) higher than 40 for downstream analysis. Supplementary Fig. 4 describes the evolution of read counts throughout the preprocessing steps. We implemented the described DNA-modification-signal positioning via custom scripts in Python version 3.7.4 [GCC 4.8.5] with the modules numpy version 1.21.5, pandas version 0.25.1 and biopython version 1.79 or, for GE and P, in Python version 3.11.6 [GCC 12.2.0] with modules numpy version 1.25.2, pandas version 2.2.2 and biopython version 1.83.
3) In DNA methylation array analysis, beta values were derived using the R package minfi version 1.50.0. The downstream analysis of beta values and their visualization were performed in Jupyter notebooks employing the modules numpy version 1.26.4, pandas version 2.2.2 and matplotlib version 3.9.0 in Python version 3.11.6 [GCC 13.2.0].

For manuscripts utilizing custom algorithms or software that are central to the research but not yet described in published literature, software must be made available to editors and reviewers. We strongly encourage code deposition in a community repository (e.g. GitHub). See the Nature Portfolio guidelines for submitting code & software for further information.

## Data

Policy information about availability of data

All manuscripts must include a data availability statement. This statement should provide the following information, where applicable:
- Accession codes, unique identifiers, or web links for publicly available datasets
- A description of any restrictions on data availability
- For clinical datasets or third party data, please ensure that the statement adheres to our policy

The raw sequencing and DNA-methylation-array data and corresponding processed data (tsv-files with called DNA-modification sites, csv-file with beta values) generated in this study are deposited in the NCBI Gene Expression Omnibus (GEO). GEO series accession code for the data of endogenous guanine oxidation in cells and AP sites in irofulven- or DMSO-exposed cells: GSE272366 [https://www.ncbi.nlm.nih.gov/geo/query/acc.cgi?acc=GSE272366]. GEO series accession code for the data of guanine oxidation in GSK-3484862- or DMSO-exposed cells, and for the data of oxidized guanines and abasic sites in plasmids with site-specific DNA modifications: GSE295218 [https://www.ncbi.nlm.nih.gov/geo/query/acc.cgi?acc=GSE295218]. GEO series accession code for the methylation data from GSK-3484862- or DMSO-exposed cells: GSE295295 [https://www.ncbi.nlm.nih.gov/geo/query/acc.cgi?acc=GSE295295]. Source Data for Figures and Extended Data Figures, Supplementary Data (Source Data for Supplementary Figures) and raw mass spectrometry data are deposited in Zenodo [https://doi.org/10.5281/zenodo.16936776].

## Research involving human participants, their data, or biological material

Policy information about studies with human participants or human data. See also policy information about sex, gender (identity/presentation), and sexual orientation and race, ethnicity and racism.

| | |
|---|---|
| Reporting on sex and gender | n/a |
| Reporting on race, ethnicity, or other socially relevant groupings | n/a |
| Population characteristics | n/a |
| Recruitment | n/a |
| Ethics oversight | n/a |

Note that full information on the approval of the study protocol must also be provided in the manuscript.

# Field-specific reporting

Please select the one below that is the best fit for your research. If you are not sure, read the appropriate sections before making your selection.

☒ Life sciences ☐ Behavioural & social sciences ☐ Ecological, evolutionary & environmental sciences

For a reference copy of the document with all sections, see nature.com/documents/nr-reporting-summary-flat.pdf

# Life sciences study design

All studies must disclose on these points even when the disclosure is negative.

| | |
|---|---|
| Sample size | Sample size determination was not based on any statistical method. We chose sample sizes based on technical challenges and throughput of each assay. |
| Data exclusions | For click-code-seq data analysis, reads with low quality and Illumina adapter read-through were removed. The counts of removed reads are provided in Supplementary Figure 4. |
| Replication | The numbers of replicated experiments are provided in figure legends, in figures and Methods. All replication attempts were successful. |
| Randomization | Cells that were exposed to a chemicals or UV and cells that were unexposed or exposed to a vehicle originated from the same parent isogenic culture, therefore the cells were randomly distributed. |
| Blinding | There was no subjective allocation for any experiments, thus blinding was not required for this study. |

# Reporting for specific materials, systems and methods

We require information from authors about some types of materials, experimental systems and methods used in many studies. Here, indicate whether each material, system or method listed is relevant to your study. If you are not sure if a list item applies to your research, read the appropriate section before selecting a response.

## Materials & experimental systems

| n/a | Involved in the study |
|---|---|
| ☒ | ☐ Antibodies |
| ☐ | ☒ Eukaryotic cell lines |
| ☒ | ☐ Palaeontology and archaeology |
| ☒ | ☐ Animals and other organisms |
| ☒ | ☐ Clinical data |
| ☒ | ☐ Dual use research of concern |
| ☒ | ☐ Plants |

## Methods

| n/a | Involved in the study |
|---|---|
| ☒ | ☐ ChIP-seq |
| ☒ | ☐ Flow cytometry |
| ☒ | ☐ MRI-based neuroimaging |

# Eukaryotic cell lines

Policy information about cell lines and Sex and Gender in Research

| | |
|---|---|
| Cell line source(s) | HAP1 wild type (Horizon Discovery, C631, RRID:CVCL_Y019)<br>U2OS wild type (Mark Rubin's lab, Department for BioMedical Research, the University of Bern)<br>BJ-5ta wild type (ATCC, CRL-4001) |
| Authentication | All cell lines were monitored through morphology and growth characteristics. Cell lines were authenticated by Microsynth AG, Switzerland. |
| Mycoplasma contamination | All cell lines were periodically tested for mycoplasma contamination and were mycoplasma negative. |
| Commonly misidentified lines<br>(See ICLAC register) | No commonly misidentified cell lines were used. |

# Plants

| | |
|---|---|
| Seed stocks | *Report on the source of all seed stocks or other plant material used. If applicable, state the seed stock centre and catalogue number. If plant specimens were collected from the field, describe the collection location, date and sampling procedures.* |
| Novel plant genotypes | *Describe the methods by which all novel plant genotypes were produced. This includes those generated by transgenic approaches, gene editing, chemical/radiation-based mutagenesis and hybridization. For transgenic lines, describe the transformation method, the number of independent lines analyzed and the generation upon which experiments were performed. For gene-edited lines, describe the editor used, the endogenous sequence targeted for editing, the targeting guide RNA sequence (if applicable) and how the editor was applied.* |
| Authentication | *Describe any authentication procedures for each seed stock used or novel genotype generated. Describe any experiments used to assess the effect of a mutation and, where applicable, how potential secondary effects (e.g. second site T-DNA insertions, mosiacism, off-target gene editing) were examined.* |
